# Interspecies Microbial Fusion and Large-Scale Exchange of Cytoplasmic Proteins and RNA in a Syntrophic *Clostridium* Coculture

**DOI:** 10.1128/mBio.02030-20

**Published:** 2020-09-01

**Authors:** Kamil Charubin, Shannon Modla, Jeffrey L. Caplan, Eleftherios Terry Papoutsakis

**Affiliations:** a Department of Chemical and Biomolecular Engineering, University of Delaware, Newark, Delaware, USA; b Delaware Biotechnology Institute, University of Delaware, Newark, Delaware, USA; c Bioimaging Center, University of Delaware, Newark, Delaware, USA; University of Washington

**Keywords:** *Clostridium ljungdahlii*, *Clostridium acetobutylicum*, syntrophy, heterologous cell fusion, hybrid cells, protein exchange, RNA exchange, anaerobic fluorescent proteins, bacteria, hybrid bacteria

## Abstract

Microbial syntrophy is universal in nature, profoundly affecting the composition and function of microbiomes. We have recently reported data suggesting direct cell-to-cell interactions leading to electron and material exchange between the two microbes in the syntrophy between Clostridium ljungdahlii and C. acetobutylicum. Here, transmission electron microscopy and electron tomography demonstrated cell wall and membrane fusions between the two organisms, whereby *C. ljungdahlii* appears to invade C. acetobutylicum pole to pole. Correlative fluorescence transmission electron microscopy demonstrated large-scale exchange of proteins. Flow cytometry analysis captured the extent and dynamic persistence of these interactions. Dividing hybrid cells were identified containing stained proteins from both organisms, thus demonstrating persistence of cells with exchanged cellular components. Fluorescence microscopy and flow cytometry of one species with stained RNA and the other tagged with a fluorescent protein demonstrated extensive RNA exchange and identified hybrid cells, some of which continued to divide, while some were in an advanced C. acetobutylicum sporulation form. These data demonstrate that cell fusion enables large-scale cellular material exchange between the two organisms. Although unanticipated and never previously reported, these phenomena are likely widely distributed in nature, have profound implications for species evolution and the function of microbial communities, and could find utility in biotechnology. They may shed new light onto little-understood phenomena, such as antibiotic heteroresistance of pathogens, pathogen invasion of human tissues, and the evolutionary trajectory and persistence of unculturable bacteria.

## Abstract (precis)

We report that two different bacterial organisms engage in heterologous cell fusion that leads to massive exchange of cellular material, including proteins and RNA, and the formation of persistent hybrid cells. The interspecies cell fusion observed here involves a syntrophic microbial system, but these heterologous cell fusions were observed even under nonstrict syntrophic conditions, leaving open the possibility that strict syntrophy may not be necessary for interspecies cell fusion and cellular material exchange. Formation of hybrid cells that contain proteins and RNA from both organisms is unexpected and unprecedented. Such fusion events are likely widely distributed in nature, but have gone undetected. The implications are profound and may shed light onto many unexplained phenomena in human health, natural environments, evolutionary biology, and biotechnology.

## INTRODUCTION

Microbial syntrophic interactions are ubiquitous in nature (1–3). Syntrophy, that is, “obligately mutualistic metabolism” (1) extends to all microbial species: prokaryotes, archaea, and eukaryotes (1, 4–6). Data from diverse microbial communities demonstrate that “metabolic dependencies are a major driver of species cooccurrence” that shapes “microbial community architecture” (4). It is broadly assumed that in syntrophies, metabolic exchanges driven by nutritional limitations occur by release of nutrients into the environment by one species and uptake by another (1, 4, 7). While syntrophy presumes a metabolic dependency, the concept may be extended to encompass nonmetabolic dependencies, such as acquisition of survival factors, including but not limited to antibiotic resistance, which could explain, for example, heteroresistance of pathogens (8) or antibiotic resistance in biofilms (9). Syntrophy may also underlie the “phenomenon” of unculturable bacteria (10).

Exchange of electrons in syntrophic and other microbial systems have been extensively studied and mechanistically explained (1, 11, 12). Beyond exchange of electrons, direct physical interactions underlying syntrophies have been visually or metabolically documented (1, 5, 6, 13). Some bacteria have been shown to form intercellular nanotubes, which facilitate the exchange of small molecules (amino acids) and cytoplasmic material (fluorescent and antibiotic-resistance proteins) between cells (14–17). In a syntrophy between the Gram-negative Desulfovibrio vulgaris and the Gram-positive Clostridium acetobutylicum, physical contact was demonstrated using scanning electron microscopy, although the interactions were not detailed at the membrane and cell wall level (6). The physical contact between the two organisms led to exchange of the dye calcein (MM of 622.55) and possibly mCherry, but fluorescence microscopy images did not permit unambiguous cellular scrutiny and did not provide interaction details.

We have recently published metabolic and transcriptional data for a glucose-fed syntrophy between the acetogen Clostridium ljungdahlii, which uses the Wood-Ljungdahl Pathway for autotrophic growth, and the solventogen C. acetobutylicum (13). C. acetobutylicum uses glucose, fructose, and other sugars to produce acetate, butyrate, butanol, acetone, ethanol, acetoin, CO_2_, and H_2_. C. ljungdahlii cannot use glucose (using only fructose among the common sugars) and cannot survive in a glucose-based culture medium in monoculture. However, in syntrophic coculture, it grows using CO_2_ and H_2_ released by C. acetobutylicum. When grown in monoculture in the basal medium (Turbo CGM) of the coculture, but supplemented with fructose and CO_2_/CO/H_2_, *C. ljungdahlii* produces only acetate and ethanol (13). In coculture, using the Turbo CGM medium supplemented with 80 g/liter glucose and 5 g/liter fructose, acetone was converted to 2-propanol (which neither organism produces in monoculture), and acetoin to 2,3 butanediol (which *C. ljungdahlii* can in principle produce alone, but did not in control cultures) (13). In coculture, 2,3-butanediol was formed without any detectable acetoin in the medium, thus suggesting direct transfer of acetoin from C. acetobutylicum to *C. ljungdahlii* (13). Furthermore, the large concentrations of 2-propanol produced in coculture combined with electron balances for the individual species’ metabolism suggested direct electron transfer (13). These phenotypes were abolished when the two organisms were separated by a permeable membrane (13). Here, we examine these interactions and the hypothesized direct transfer of materials between the two microbes.

## RESULTS

### Unanticipated cell fusion events between C. acetobutylicum and C. ljungdahlii.

**FIG 1:**
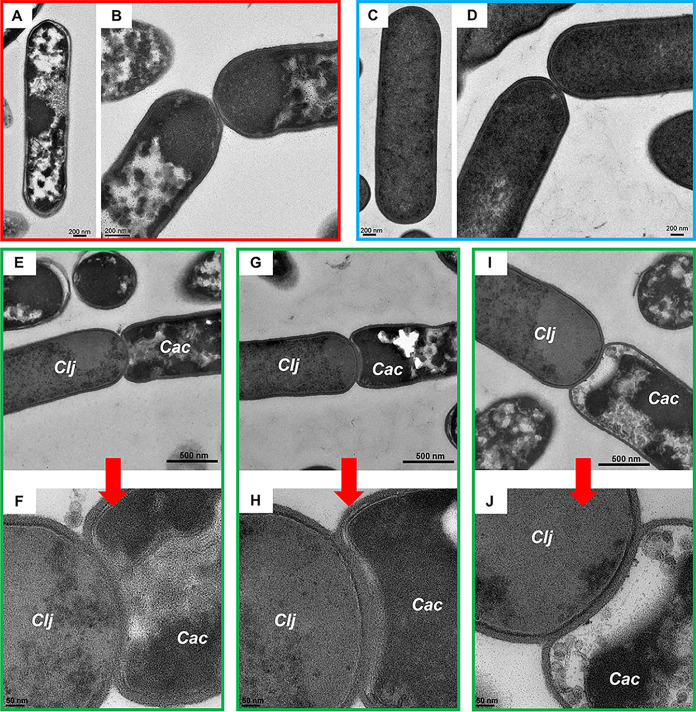
Thin-section TEM of C. acetobutylicum and C. ljungdahlii monocultures and coculture. (A and B) TEM images of C. acetobutylicum after 24 h of monoculture. (C and D) *C. ljungdahlii* after 48 h of monoculture. After 24 h, C. acetobutylicum cells differentiate as part of their sporulation program forming complex cellular structures, including granulose, which appear as irregular and electron-translucent intracellular regions by TEM. In comparison, *C. ljungdahlii* did not differentiate even after 48 h of growth. (E to J) After 24 h, coculture samples contained differentiated C. acetobutylicum (Cac) cells and vegetative *C. ljungdahlii* (Clj) cells. Thin-section TEM: differentiating C. acetobutylicum cells (right, lower right) can be easily distinguished from the vegetative homogeneous texture of *C. ljungdahlii* cells (left, upper left). C. acetobutylicum and *C. ljungdahlii* formed fusion events where their cell walls and cell membranes fused in the coculture. No cell fusion was observed in the monoculture. Red arrows indicate closeup (panels F, H, and J) images of the fusion events of panels E, G, and I, respectively.

Cells from cocultures and monoculture controls were imaged using TEM. C. acetobutylicum monoculture and cocultures were grown in Turbo CGM medium supplemented with 80 g/liter glucose and 5 g/liter fructose, while *C. ljungdahlii* monocultures were grown in the same medium supplemented with 5 g/liter of fructose and 20 lb/in^2^ of H_2_/CO_2_ (80/2%) gas mixture as described (13). After 24 h in culture, as part of its sporulation program (18–20) C. acetobutylicum developed complex cytoplasmic structures (Fig. 1A and B), which in TEM appear as areas of low electron density (19–21). In contrast, *C. ljungdahlii* monocultures do not differentiate, maintaining a homogeneous electron-dense vegetative cytoplasm even after 48 h in culture (Fig. 1C and D) (13, 22). In comparison, cocultures contained differentiating C. acetobutylicum cells and vegetative *C. ljungdahlii* cells, with morphologies consistent with those of monocultures. C. acetobutylicum and *C. ljungdahlii* in coculture interacted at the poles, where *C. ljungdahlii* appeared to invade the C. acetobutylicum cytoplasm (Fig. 1E to J and Fig. S1). In these previously unknown and unanticipated interactions, the outer peptidoglycan walls of *C. ljungdahlii* and C. acetobutylicum fused. The extent of interaction varied among the captured events. In Fig. 1F, the inner membranes of the organisms appear fused, in contrast to the image of Fig. 1J, where the membranes of *C. ljungdahlii* and C. acetobutylicum appear intact. Figure 1H shows an intermediate stage of cell fusion, where C. acetobutylicum’s distinct membrane apparently disappears at the fusion site. After 24 h of coculture, fusion events represented ca. 10% of all cells in samples examined via TEM; 45 cells formed fusions out of 447 cells in the examined section plane. No fusion events were observed in monoculture controls (Fig. 1A to D), where cells of the same organism touching at the poles maintained distinct peptidoglycan walls and inner membranes. We have examined over 200 sets of TEM images of C. acetobutylicum monocultures over the years and have never observed fusion events observed in the coculture. Binary cell division in C. acetobutylicum and *C. ljungdahlii* appears very different from fusion events: both cells are in a vegetative state, and there is no cytoplasmic invasion (Fig. S2). Thus, the cell fusion and cytoplasmic invasion observed between C. acetobutylicum and *C. ljungdahlii* are unique to the syntrophic coculture.

10.1128/mBio.02030-20.1 FIG S1 Cell fusion between C. acetobutylicum and *C. ljungdahlii* in the coculture. Additional TEM images of the C. acetobutylicum*-C. ljungdahlii* fusion after 24 h of coculture. Cocultures contained sporulating C. acetobutylicum cells (with electron-translucent regions) and vegetative *C. ljungdahlii* cells (with homogeneous electron-dense cytoplasm). In each example shown, *C. ljungdahlii* appears to invade C. acetobutylicum*’*s cytoplasm, which leads to fusion of cell membrane and cell walls of both organisms. Red arrows indicate closeup (panels B, D, and F) images of the fusion events of panels A, C, and E, respectively. Download FIG S1, DOCX file, 1.3 MB. Copyright © 2020 Charubin et al. 2020 Charubin et al. This content is distributed under the terms of the Creative Commons Attribution 4.0 International license.

10.1128/mBio.02030-20.2 FIG S2 TEM imaging of *C. ljungdahlii* and C. acetobutylicum cell division in monocultures. TEM images of vegetative *C. ljungdahlii* cells undergoing cell division. (A) Pinching at the midpoint to begin cell division. (B) Beginning of septum formation. (C) Septum is fully formed. (D) Daughter cells fully identifiable with distinct cell membranes and walls. No cytoplasmic invasion is present, compared to cell fusion events of Fig. 1 and Fig. S1 in the coculture. (E and F) C. acetobutylicum undergoes cell division in the same fashion and does not resemble cell fusion observed in the coculture. Download FIG S2, DOCX file, 0.8 MB. Copyright © 2020 Charubin et al. 2020 Charubin et al. This content is distributed under the terms of the Creative Commons Attribution 4.0 International license.

**FIG 2:**
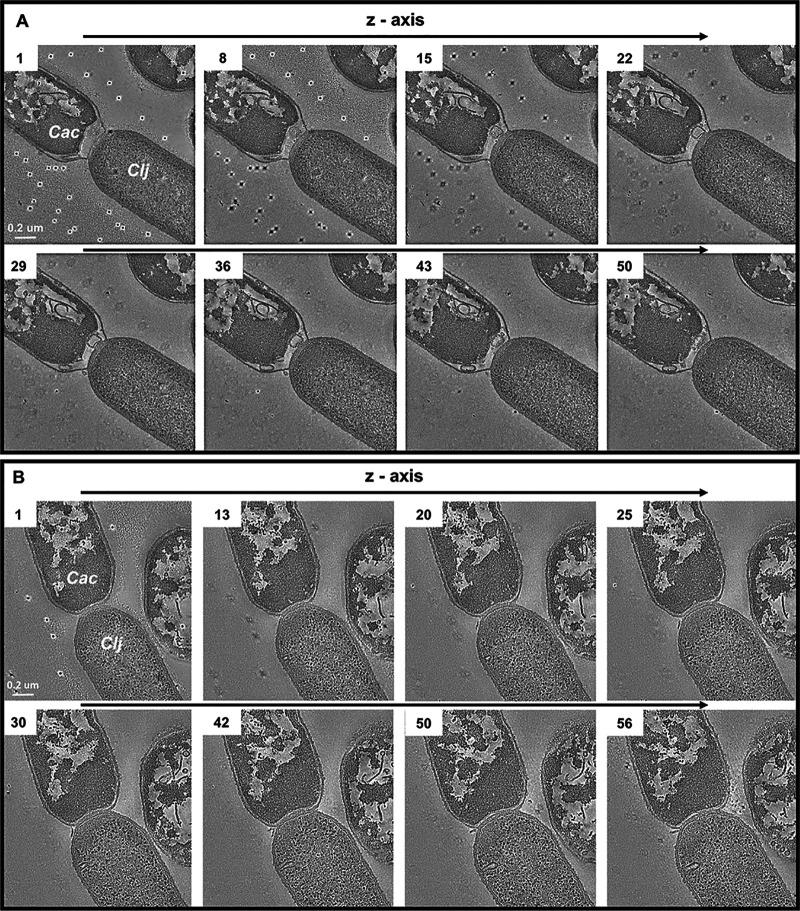
Electron tomography of C. acetobutylicum and C. ljungdahlii fusion pairs observed in the coculture. (A) TEM tomography: differentiating C. acetobutylicum cells (top left, Cac)) can be easily distinguished from the vegetative homogeneous texture of *C. ljungdahlii* cells (bottom right, Clj). The cell fusion between the two organisms persists through the entire depth of the tomography (∼150 nm). Dark marks in the background are gold fiducial markers used for aligning the image series. (B) TEM tomography of another C. acetobutylicum*-C. ljungdahlii* fusion pair.

We also examined cell fusion via electron tomography, where three-dimensional information is obtained by tilting the sample stage and recording images at each incremental tilt angle (see the Materials and Methods). Tomography was performed on 150-nm-thick sections of resin-embedded samples. Figure 2A shows eight images (from a series of 58) to examine the spatial progression of the fusion event. As in Fig. 1E to J, the top left cell contains complex cytoplasmic structures characteristic of differentiating C. acetobutylicum cells, while the bottom right cell displays the homogeneous electron density expected for *C. ljungdahlii* cells after 24 h of culture. The first 40 images in the series display the *C. ljungdahlii*-to-C. acetobutylicum invasion process. Since the tomogram captures only one-third of the total cell volume, the tomographic series contains information from approximately the middle of the fusion event through the bottom. Figure 2B shows another tomography series of a different fusion pair. In the tomographic images of Fig. 2A and the TEM images of Fig. 1E to J and Fig. S1, C. acetobutylicum’s cytoplasmic contents appear retracted from the pole as if the cytoplasmic membrane is pulled away from the cell wall.

We note that in all coculture experiments, the basal Turbo CGM medium contained 80 g/liter glucose and 5 g/liter fructose. Fructose is used by both C. acetobutylicum and *C. ljungdahlii* and is not exhausted until about 50 h of coculture (13). The observed cell fusion events of Fig. 1 and 2 were observed at 24 h, and as shown below, even earlier. However, the same coculture phenotype is observed with significantly lower starting fructose concentration (0.8 g/liter) in the medium, and when no fructose is present in the system, such as when the coculture is passaged into a medium with glucose alone (13). In the latter case, *C. ljungdahlii* depends solely on CO_2_ and H_2_ released by C. acetobutylicum for growth and survival. Our data then show that the presence of fructose does not affect the fusion events and the resulting cellular material exchange between the two organisms discussed below. However, the coculture system is syntrophic in nature even in the presence of fructose. This is because *C. ljungdahlii* grows much slower and reaches lower cell densities (optical density [OD] of ∼1) when grown on fructose alone, or CO_2_/H_2_ alone (13). When both fructose and gases are present, *C. ljungdahlii* performs much better in mono- and cocultures. We have also shown that even in the presence of fructose in the medium, *C. ljungdahlii* consumes almost all the H_2_ and most of the CO_2_ released by C. acetobutylicum in the coculture (13).

### Large-scale exchange of proteins between the two organisms.

**FIG 3:**
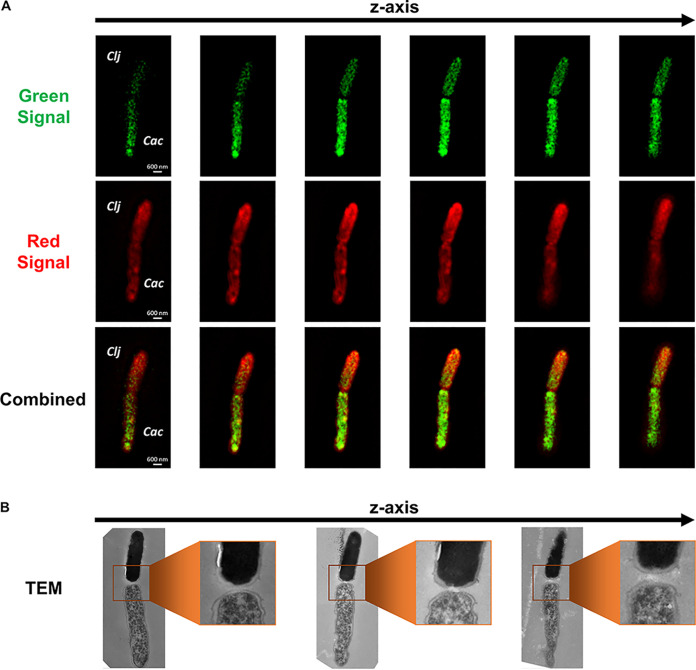
Correlative confocal and electron microscopy of the C. acetobutylicum*-C. ljungdahlii* fusion event and the exchange of protein between the two organisms. (A) Fluorescence SR-SIM imaging of a cell fusion event between C. acetobutylicum*-*FAST (Cac) and Red-labeled *C. ljungdahlii* (Clj) shows the exchange of proteins between the organisms. The green protein from C. acetobutylicum-FAST (FAST bound to HMBR) is diffusing into the *C. ljungdahlii*’s cytoplasm. Red-stained proteins (CellTracker Deep Red) from *C. ljungdahlii* display a reverse gradient diffusing into C. acetobutylicum*-*FAST’s cytoplasm. The identity of each cell was based on the strength of the fluorescence signal. (B) Correlative TEM of the same fusion pair revealed the characteristic ultrastructure of each cell and confirmed the initial identification. The top cell was *C. ljungdahlii* with homogeneous cytoplasm, while the bottom cell was a differentiated C. acetobutylicum-FAST cell. The middle TEM section shows the interaction at the poles between the two strains consistent with the TEM images of Fig. 1 and Fig. S1.

Beyond the exchange of small molecules (acetone, acetoin) we have previously demonstrated (13), would cell fusion facilitate exchange of other cellular material, such as proteins? To examine this hypothesis, it was necessary to label each organism with a different fluorescent tag. To effectively tag C. acetobutylicum cells, we adapted the O_2_-independent fluorescent protein FAST (23), which fluoresces green when exposed to ligand HMBR (24). To explore large-scale exchange of proteins, we used the protein-staining CellTracker Deep Red dye, which produces far-red fluorescence not overlapping with FAST emission. Deep Red (denoted Red below) passes freely through cell membranes. Once inside cells, its succinimidyl ester group reacts with protein amine groups, rendering the dye membrane impermeant. It is thus retained in cells through several generations; the dye is transferred to daughter cells, but not to adjacent cells in a population (25). Control experiments confirmed this, where C. acetobutylicum-ZapA-FAST cells cultured in spent medium from Red-labeled *C. ljungdahlii* cells (and vice versa) did not acquire any red fluorescence (Fig. S3). Samples of C. acetobutylicum-FAST and Red-labeled *C. ljungdahlii* cells in coculture were examined using correlative confocal-transmission electron microscopy. Superresolution structured-illumination microscopy (SR-SIM; see the Materials and Methods) identified interacting C. acetobutylicum-*C. ljungdahlii* pairs and visualized the localization of fluorescent proteins (FAST protein in C. acetobutylicum, and Red-labeled proteins in *C. ljungdahlii*). In the fusion event of Fig. 3A, the fluorescence signal from the Red-labeled *C. ljungdahlii* proteins was stronger in the top cell displaying a “diffusion” gradient into the bottom cell. The green signal (FAST protein) from C. acetobutylicum displayed an opposite gradient, diffusing into the Red *C. ljungdahlii* cell (Fig. 3A). Based on the intensity of red and green fluorescence, the top cell was arguably Red-labeled *C. ljungdahlii*, while the bottom was a green-labeled C. acetobutylicum-FAST cell (Fig. 3A). Correlative TEM of the same fusion pair confirmed that the top cell was a “homogeneous” *C. ljungdahlii* cell, while the bottom was a differentiating C. acetobutylicum-FAST cell (Fig. 3B). Their peptidoglycan walls appeared fused at the poles (Fig. 3B), similar to the fusion events of Fig. 1E to J and Fig. S1. The red fluorescence gradient (*C. ljungdahlii* proteins diffusing into the C. acetobutylicum cytoplasm) indicated large-scale exchange of proteins between the two organisms (Fig. 3A). Similarly, the green-fluorescence gradient demonstrated movement of the heterologous FAST protein from C. acetobutylicum-FAST into *C. ljungdahlii* (Fig. 3A). Although TEM images captured only a small fraction of the cell depth (Fig. 3B), protein transfer between the two organisms occurred throughout the whole cell depth, as shown by the Z-stack series of fluorescence-microscopy images (Fig. 3A).

10.1128/mBio.02030-20.3 FIG S3 Control experiments to confirm that protein exchange occurs only through C. acetobutylicum-*C. ljungdahlii* fusion. (A) Experimental set up. Monocultures of C. acetobutylicum-ZapA-FAST and Red *C. ljungdahlii* (Deep Red dye) were grown for 24 h. Entire cultures were pelleted and supernatants filtered with a 0.2-μm filter. “Red” medium was the filtered spent medium from Red *C. ljungdahlii* culture; “Green” medium was the filtered spent medium from C. acetobutylicum*-*ZapA-FAST culture. The Red *C. ljungdahlii* pellet was placed in the Green medium, while the C. acetobutylicum-ZapA-FAST pellet was placed in the Red medium. Cells in spent media were monitored using flow cytometry for 24 h to detect if any fluorescent material that may have leaked into the spent medium is possibly taken up by the other organism. (B) Flow cytometry analysis of cultures described in (A). Red *C. ljungdahlii* cells placed in Green medium did not develop any green fluorescence after 24 h in culture. C. acetobutylicum-ZapA-FAST cells placed in Red medium did not develop any red fluorescence after 24 h of culture. Therefore, there was no fluorescent material in the spent media that could be up taken by cells to produce false double-positive cells. Thus, protein exchange can only occur through C. acetobutylicum*-C. ljungdahlii* fusion. Download FIG S3, DOCX file, 1.1 MB. Copyright © 2020 Charubin et al. 2020 Charubin et al. This content is distributed under the terms of the Creative Commons Attribution 4.0 International license.

Since Gram^+^ cells secrete many proteins containing an N-terminal signal peptide (26, 27), could the observed protein exchange be limited to exported proteins? As the heterologous FAST protein contains no signal peptide for protein export (28, 29), these data suggest that FAST is transported from C. acetobutylicum to *C. ljungdahlii*, but the red signal in C. acetobutylicum cells may just represent Red *C. ljungdahlii* proteins tagged for export. To examine this further, we carried out similar experiments using another suitable dye (green fluorescent protein dye CellTrace CFSE) to label C. acetobutylicum proteins. The data (Fig. S4) suggest transport of green-tagged C. acetobutylicum proteins into *C. ljungdahlii*. Even if one assumes that only secreted proteins are being exchanged, since the C. acetobutylicum protein secretome is large (29), these data suggest that there is large-scale protein exchange between the two organisms.

10.1128/mBio.02030-20.4 FIG S4 Protein exchange after 20 h in coculture between red-labeled WT *C. ljungdahlii* (labeled with CellTracker Deep Red) and green-labeled WT C. acetobutylicum (labeled with CellTrace CFSE). Images acquired by SR Airyscan confocal microscopy. Many cells exchanged proteins as assessed by exchange of green or red-labeled proteins, but several did not. (A to C) Single long green “pure” C. acetobutylicum cell, probably undergoing cell division. (D to F) A long red “pure” *C. ljungdahlii* cell probably undergoing cell division. (G to I) Pairs of cells similar to those of Fig. 1 and 3 of the main text, where a Red *C. ljungdahlii* cell fused with a green C. acetobutylicum cell, thus exchanging proteins. We also observed several double-positive hybrid cells containing equally distributed fluorescent signals at different cell cycle stages. (J to L) Single hybrid cell. (M to O) Elongated hybrid cell. (P to R) Long hybrid cell, undergoing cell division. Download FIG S4, DOCX file, 0.8 MB. Copyright © 2020 Charubin et al. 2020 Charubin et al. This content is distributed under the terms of the Creative Commons Attribution 4.0 International license.

### Formation of hybrid C. acetobutylicum*-*C. ljungdahlii cells.

**FIG 4:**
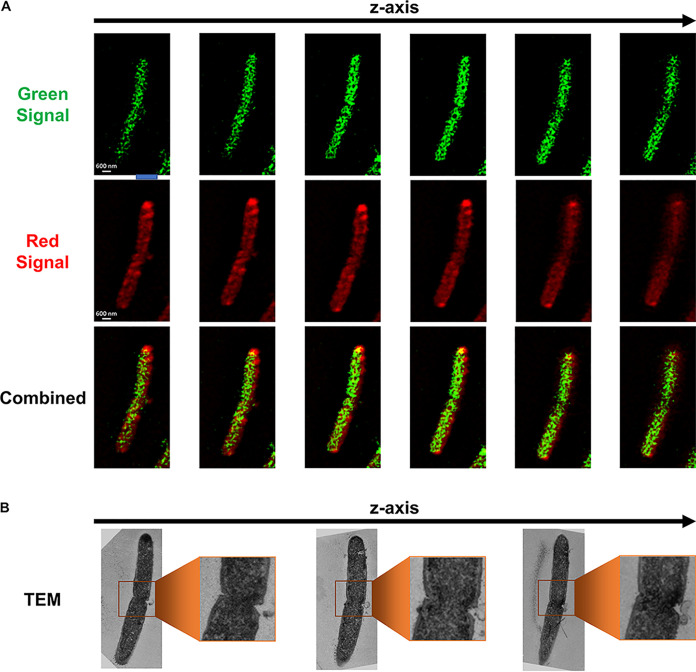
Correlative confocal and electron microscopy captures hybrid C. acetobutylicum*-C. ljungdahlii* cells which form in the coculture through cell fusion and material transfer. (A) Correlative SR-SIM imaging of a hybrid C. acetobutylicum*-C. ljungdahlii* cell undergoing division. Both cells display equally distributed red (*C. ljungdahlii* protein bound to CellTracker Deep Red) and green (HMBR bound to FAST) fluorescence. An equal distribution of fluorescent material was the result of a hybrid cell (containing protein from both organisms) undergoing cell division. (B) Correlative TEM of the same cell shows that the ultrastructure of both cells appears to be a hybrid of the two organisms.

What happens to each cell after fusion and exchange of proteins? In the pair of Fig. 4A, both cells displayed green and red fluorescence, but the intensity of each signal appeared homogeneous throughout both cells, making it impossible to identify each cell based on fluorescence. This assessment was valid for the entire Z-stack series of this pair (Fig. 4A). Correlative TEM images revealed that the two cells had the same ultrastructure appearance (Fig. 4B), which resembled neither C. acetobutylicum (lack of large uneven translucent regions; Fig. 1A and B), nor *C. ljungdahlii* (not fully homogeneous cytoplasm; Fig. 1C and D). Both cells appeared to be hybrids of C. acetobutylicum and *C. ljungdahlii* morphologies. Significantly, the contact point between the cells was not consistent with the cell fusion events (Fig. 1E to J, Fig. 2, and Fig. 3), and instead resembled the cell membrane invagination occurring during cell division in pure C. acetobutylicum and *C. ljungdahlii* cells (Fig. S2). This is apparently a dividing *hybrid cell*, defined as a cell that contains uniformly distributed proteins from both organisms. The term hybrid here is used to distinguish such cells from cells exchanging proteins while retaining the morphological characteristics of one or the other cell type. Accordingly, the cells of Fig. 1 and 3 are not hybrid cells. Therefore, the cells shown in Fig. 4 were likely the progeny of a dividing C. acetobutylicum-FAST cell which had fused with a Red *C. ljungdahlii* cell and acquired its proteins (or vice versa). The fusion and protein exchange resulted in formation of hybrid cells, containing protein from both organisms. Such cells apparently divided and continued to grow. Such events are surprising and unprecedented.

### Coculture of C. acetobutylicum-FAST-ZapA and *C. ljungdahlii*-Halo strains confirms extensive protein exchange.

**FIG 5:**
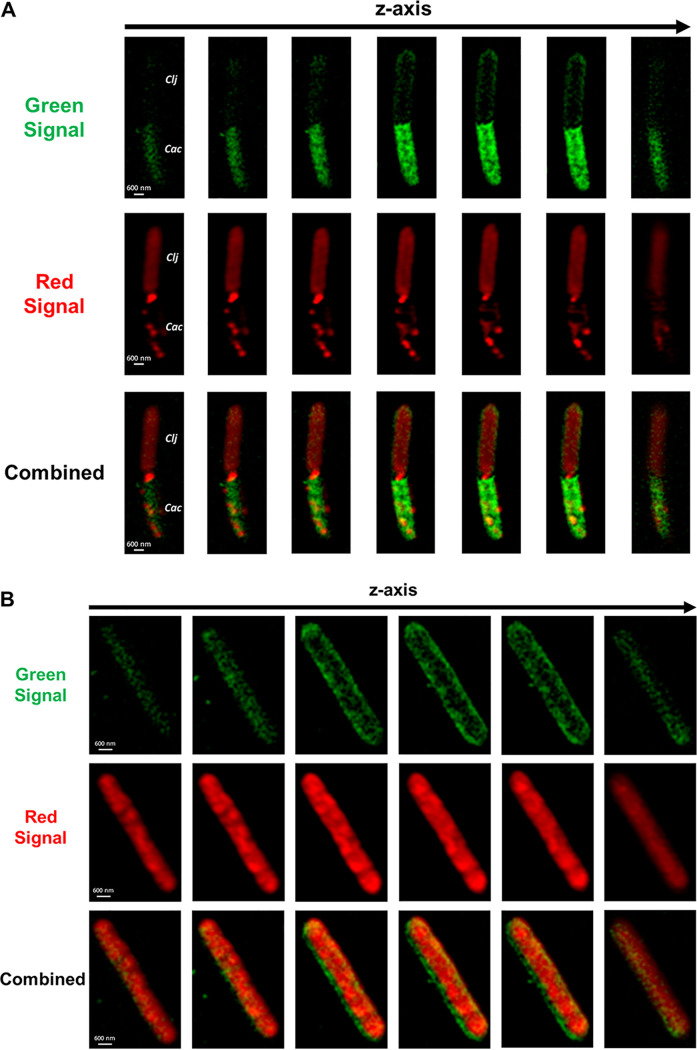
Protein exchange between the C. acetobutylicum-ZapA-FAST and *C. ljungdahlii*-Halo strains in the syntrophic coculture. (A) Fusion event between *C. ljungdahlii*-Halo and C. acetobutylicum-ZapA-FAST captured using SR-SIM imaging. Strains were identified based on the strength of each fluorescent signal. Red signal is from Janelia Fluor 646 ligand bound to HaloTag in *C. ljungdahlii*-Halo strain; green signal is from HMBR ligand bound to ZapA-FAST protein in C. acetobutylicum. This fusion pair is similar to the one shown in Fig. 3. (B) A long (ca. 5 μm) C. acetobutylicum*-C. ljungdahlii* hybrid cell preparing for, or undergoing, cell division. The cell contains equally distributed red and green fluorescent proteins. The cell is almost double the length of a single C. acetobutylicum or *C. ljungdahlii* cell (ca. 2 μm long).

To further study the protein exchange, and show that both organisms can exchange proteins that are not tagged for export, we used the C. acetobutylicum-ZapA-FAST strain, which expresses a fluorescent fusion protein of the FAST protein fused to the C-terminal end of the cell division ZapA protein (23). We also constructed a *C. ljungdahlii*-Halo strain expressing the HaloTag protein (Fig. S5). HaloTag does not fluoresce on its own; instead, it covalently binds to a variety of fluorescent ligands (30, 31). We used Janelia Fluor 646 ligand to label the *C. ljungdahlii*-Halo strain, which produced far-red fluorescence orthogonal to the green FAST fluorescence. *C. ljungdahlii*-Halo was cocultured with C. acetobutylicum-ZapA-FAST and imaged after 20 h. The Z-stack series of Fig. 5A shows interactions similar to the cell fusion shown in Fig. 3, where the top cell displayed strong red fluorescence, thus being identified as a *C. ljungdahlii*-Halo cell, while the bottom cell had strong green fluorescence, thus being identified as a C. acetobutylicum-ZapA-FAST cell. In comparison, Fig. 5B shows an elongated cell (5 μm in length, twice the typical length of a single cell) containing equally distributed red and green fluorescence. This is arguably a C. acetobutylicum*-C. ljungdahlii* hybrid cell, containing tagged proteins from both organisms equally distributed throughout the cytoplasm, similar to the hybrid cell of Fig. 4. Based on its length, this hybrid cell (Fig. 5B) was likely preparing for cell division, and as a result it is twice as long as single *Clostridium* cells. These data suggest that protein exchange between the two organisms is not restricted to natively secreted proteins and, significantly, hybrid cells containing proteins from both organisms continue to grow and divide.

10.1128/mBio.02030-20.5 FIG S5 *C. ljungdahlii*-Halo and C. acetobutylicum-Halo strains expressing the HaloTag protein used for fluorescent labeling of cells in the coculture. (A) Fluorescent labeling of WT *C. ljungdahlii* cells with the HaloTag TMR Direct ligand. (B) Fluorescent labeling of *C. ljungdahlii-*Halo strain with same ligand. (D) Fluorescent labeling of WT C. acetobutylicum with the same ligand. (E) Fluorescent labeling of C. acetobutylicum*-*Halo strain with the same ligand. Orange fluorescence was measured by flow cytometry. WT *C. ljungdahlii* and C. acetobutylicum cells do not fluoresce after labeling with TMR Direct ligand. *C. ljungdahlii-*Halo and C. acetobutylicum*-*Halo strains show high intensity orange fluorescence when labeled with TMR Direct ligand. (C) SR-SIM imaging of *C. ljungdahlii-*Halo strain labeled with the far-red Janelia Fluor646 ligand. (F) SR-SIM imaging of C. acetobutylicum-Halo strain labeled with Janelia Fluor646 ligand. The *C. ljungdahlii*-Halo and C. acetobutylicum-Halo cells showed strong far-red fluorescence. Download FIG S5, DOCX file, 1.2 MB. Copyright © 2020 Charubin et al. 2020 Charubin et al. This content is distributed under the terms of the Creative Commons Attribution 4.0 International license.

### Dynamics of coculture interactions using flow cytometry.

**FIG 6:**
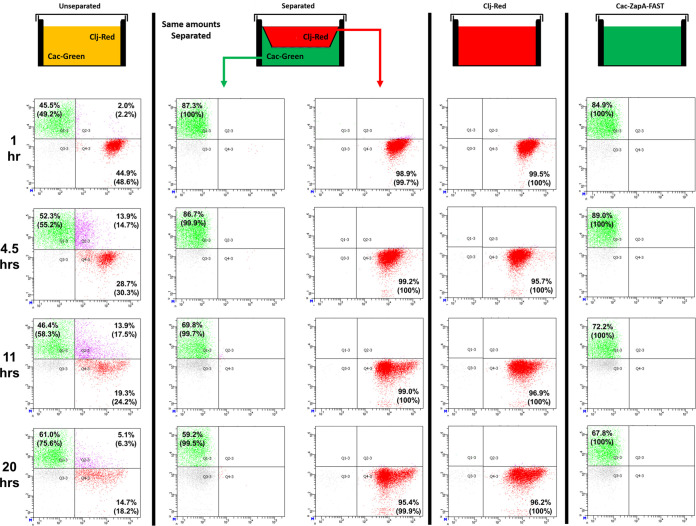
Transwell cultures demonstrate that C. acetobutylicum-ZapA-FAST and *C. ljungdahlii*-DeepRed do not exchange proteins when physically separated. Comparison of transwell unseparated versus separated cocultures (and monocultures of) between C. acetobutylicum-ZapA-FAST and wild-type *C. ljungdahlii* labeled with the CellTracker Deep Red dye over 20 h. The 100-nm membranes separating the two organisms allowed exchange of small molecules, but prevented C. acetobutylicum and *C. ljungdahlii* cells from physically interacting. In unseparated coculture, *C. ljungdahlii* and C. acetobutylicum cells interacted and exchanged fluorescent material, producing double-positive (labeled) cells. In separated coculture, no double-positive (labeled) cells developed. The Deep Red dye was fully retained in *C. ljungdahlii* cells and did not leak out over time, and C. acetobutylicum cells remained green without any red contamination. Pure Red *C. ljungdahlii* or C. acetobutylicum-ZapA-FAST control monocultures retained the red or green color throughout the time course, respectively. The values in parentheses represent normalized fluorescent populations in each sample, determined by dividing each fluorescent percentage by the total fluorescent population, i.e., the sum of the green, red, and double-positive cells. Green C. acetobutylicum-ZapA-FAST cells were not 100% labeled from the beginning, as has been documented (23). Quadrangle gates were determined using pure green- and red-fluorescing cells (Fig. S7).

We used flow cytometry to examine protein-exchange dynamics in the coculture of C. acetobutylicum-ZapA-FAST and Red *C. ljungdahlii*. Unlabeled cells were the result of fluorescent cells with a signal too weak to detect. At 1 h, there was an equal number of green C. acetobutylicum-ZapA-FAST (49.2%) and red-labeled *C. ljungdahlii* (48.7%), with few double-positive cells (Fig. S6). The two organisms appear to form many fusion events (double-positive cells) during the first 11 h, reaching 17.5% of the population. These numbers, however, underestimate the real population of double-positive cells. As the coculture grew, the fraction of double-positive cells was reduced at the expense of the green C. acetobutylicum population, and this was accompanied by a drastic reduction of the red *C. ljungdahlii* population. The latter is in contrast to the control cultures (red and green cells separated, and control containing red cells alone; Fig. 6), which display a strong red *C. ljungdahlii* population at well past 20 h. In other words, there is no loss of red fluorescent proteins in *C. ljungdahlii* due to its slow growth. This shows that *C. ljungdahlii* transfers the red-labeled proteins in the coculture to C. acetobutylicum, which grows faster than *C. ljungdahlii* (13). This results in dilution and replacement of red-labeled proteins within *C. ljungdahlii* cells with green ZapA-FAST acquired from C. acetobutylicum, which over time will be detected as pure green cells, instead of double-positive hybrid cells. As a result, the fraction of double-positive cells decreased after 11 h and disappeared after 27 h. To ensure that the observed exchange of fluorescent proteins was a result of direct cell contact, we performed control cocultures in transwell plates, where C. acetobutylicum-ZapA-FAST (bottom) was separated from Red *C. ljungdahlii* (top) with a permeable membrane (Fig. 6). The 100-nm pore membrane allows small molecules and proteins to move between the two compartments, while preventing direct cell-to-cell interaction between the two organisms. When separated, no double-positive cells were observed in either compartment. As a result, C. acetobutylicum and *C. ljungdahlii* could not exchange proteins and form double-positive hybrid cells. Furthermore, as we have shown previously, the physical separation of the two organisms abolished the unique coculture phenotype (production of isopropanol and 2,3-butanediol) (13). Therefore, the unique metabolic phenotype of the coculture must be the result of large-scale protein exchange between the two organisms. As a result of protein exchange, cells could contain enzymes from both C. acetobutylicum and *C. ljungdahlii*, which would enable the formation of nonnative metabolic pathways in these cells and the production of nonnative products like isopropanol and 2,3-butanediol (13). Significantly, the absence of double-positive cells in the separated system demonstrated that fluorescent ZapA-FAST or red-dye-labeled *C. ljungdahlii* proteins were not released to stain other cells and give rise to false double-positive cells. Without the separating permeable membrane, double-positive cells were formed as in the larger-scale coculture (Fig. S6).

10.1128/mBio.02030-20.6 FIG S6 Flow-cytometric examination of a coculture between green C. acetobutylicum-ZapA-FAST cells and wild-type (WT) *C. ljungdahlii* cells labeled with CellTracker Deep Red. Green (vertical) and red (horizontal) axes represent the intensity of green and red fluorescence. Gates for green (Q1-3), red (Q4-3), and double-positive (Q2-3) quadrangles (Q) were set based on fluorescence of individual strains and unlabeled cells (Fig. S7). Percentages represent the fraction of the total cell population in each quadrangle. Numbers in parentheses represent the normalized fraction of fluorescent cells only, obtained by dividing the cells in each fluorescent population by the total number of fluorescent cells. Unlabeled cells were the result of fluorescent cells with a signal too weak to detect. At one hour, there was an equal number of green C. acetobutylicum-ZapA-FAST (49.2%) and red-labeled *C. ljungdahlii* (48.7%) cells, with few double-positive cells. The two organisms appear to form many fusion events (double-positive cells) during the first 11 h, until reaching 17.5% of the population. The fraction of double-positive cells decreased after 11 h and disappeared after 27 h. Download FIG S6, DOCX file, 1.9 MB. Copyright © 2020 Charubin et al. 2020 Charubin et al. This content is distributed under the terms of the Creative Commons Attribution 4.0 International license.

10.1128/mBio.02030-20.7 FIG S7 Setting flow cytometry gates for analysis of cocultures between C. acetobutylicum-ZapA-FAST and WT *C. ljungdahlii* labeled with CellTracker Deep Red. Gates 1-3, 2-3, 3-3, and 4-3 contain green, double-positive (labeled), unlabeled, and red cells, respectively. (A and B) Gate 3-3 for unlabeled cells was set using WT *C. ljungdahlii* cells with no ligand and the HMBR ligand (for the FAST protein), respectively. (C) Gate 4-3 for pure red cells was set using pure *C. ljungdahlii* cells labeled with CellTracker DeepRed. (D) Gate 1-3 for pure green cells was set using pure C. acetobutylicum-ZapA-FAST cells mixed with HMBR ligand. Accordingly, gate 2-3 contains any cells that acquired both fluorescent proteins. The green (vertical) and red (horizontal) axes represent the intensity of green and red fluorescent signals, respectively. Green C. acetobutylicum-ZapA-FAST cells are not 100% labeled due to the low fluorescence of a cell subpopulation, as has been documented (23). Download FIG S7, DOCX file, 1.0 MB. Copyright © 2020 Charubin et al. 2020 Charubin et al. This content is distributed under the terms of the Creative Commons Attribution 4.0 International license.

### Cell fusion leads to exchange of RNA in the syntrophic coculture.

**FIG 7:**
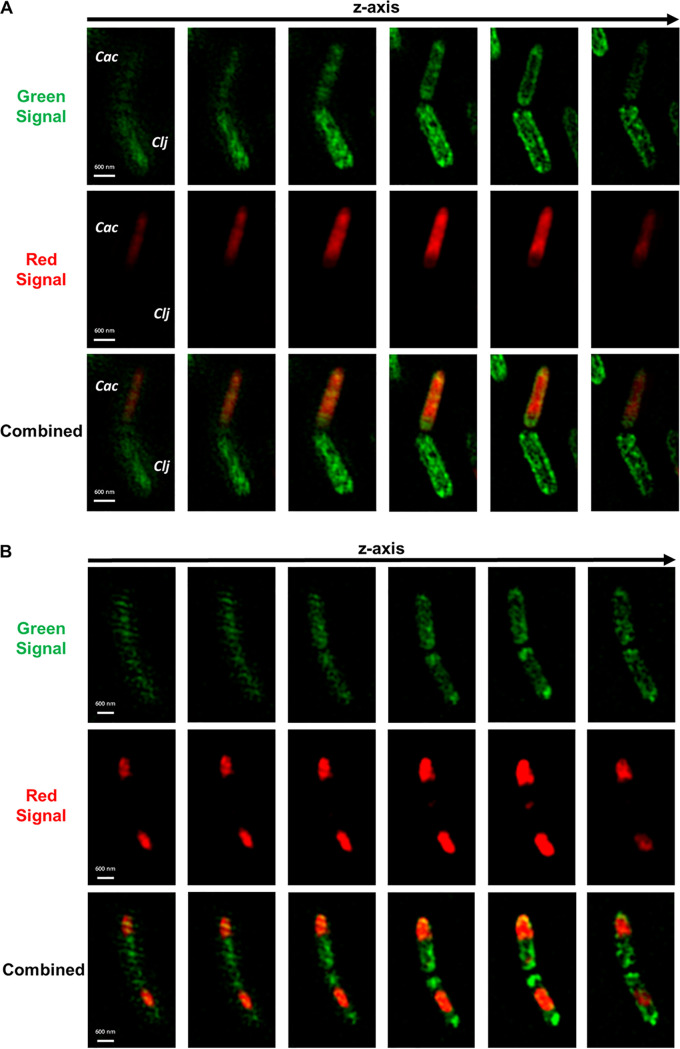
Cell-to-cell fusion facilitates RNA exchange between red C. acetobutylicum-Halo and WT *C. ljungdahlii* labeled green with SYTO RNASelect dye. The red signal came from Janelia Fluor 646 ligand bound to the HaloTag in C. acetobutylicum-Halo strain. The green signal came from RNASelect dye bound to RNA in *C. ljungdahlii* cells. Images were collected at different coculture time points in Z-stack mode using SR-SIM. (A) Cell fusion between C. acetobutylicum-Halo (top, Cac) and *C. ljungdahlii* with labeled RNA (bottom, Clj) similar to Fig. 3 at 4 h. (B) Cell fusion between two cells after 9 h of coculture. In this fusion event the cells fully exchanged the fluorescent RNA and protein. As a result, the fluorescent RNA and protein are equally distributed in each cell, making them hybrid cells.

**FIG 8:**
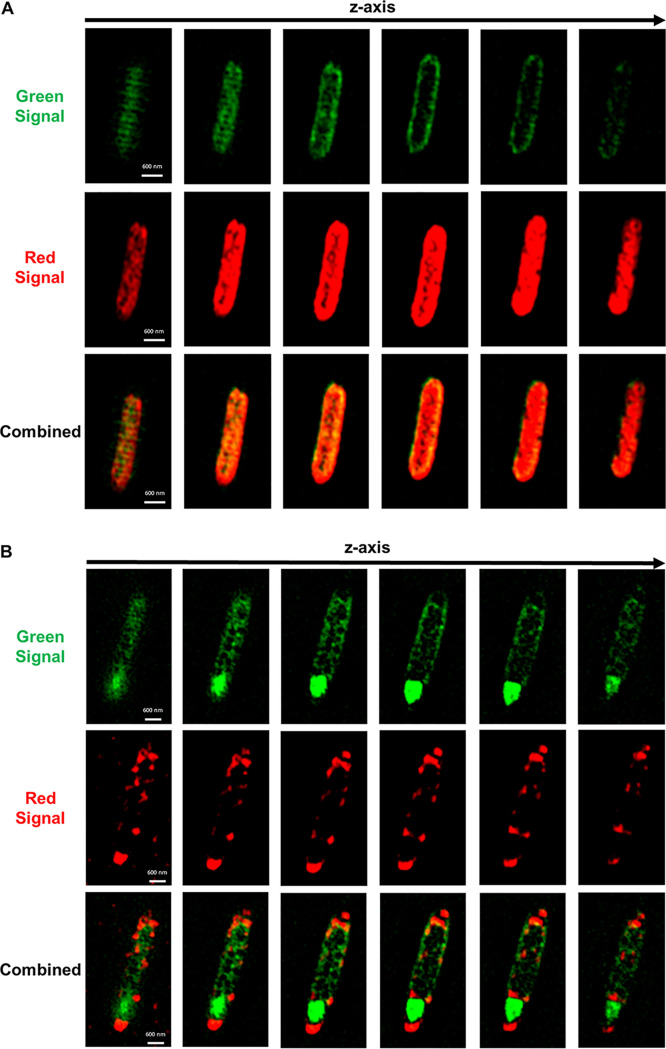
Hybrid cells in the coculture of red C. acetobutylicum-Halo and WT *C. ljungdahlii* labeled green with SYTO RNASelect dye. The red signal came from Janelia Fluor 646 ligand bound to the HaloTag in C. acetobutylicum-Halo strain. The green signal came from RNASelect dye bound to RNA in *C. ljungdahlii* cells. Images were collected at different coculture time points in Z-stack mode using SR-SIM. (A) A single hybrid cell observed at 4 h of coculture. The cell contains red protein and green RNA, which are uniformly distributed throughout the cell. (B) A single hybrid cell observed at 20 h of coculture. The cell contains both red and green fluorescent material, and displays the clostridial sporulation form associated with the sporulation mechanism in C. acetobutylicum.

Since C. acetobutylicum and *C. ljungdahlii* were found to exchange proteins in large scale, it was likely that they exchanged other cytoplasmic material. To test whether the two organisms can exchange RNA through the observed cell fusion, we labeled wild-type *C. ljungdahlii* with the SYTO RNASelect dye, which fluoresces green only when bound to RNA molecules. *C. ljungdahlii* cells with labeled RNA were cocultured with the C. acetobutylicum-Halo strain expressing the HaloTag protein (Fig. S5). Thus, in this coculture, the green signal was produced by the RNASelect dye bound to *C. ljungdahlii*’s RNA, while the red color was produced by the HaloTag protein bound to Janelia 646 ligand in C. acetobutylicum-Halo strain. Starting at 4 h of coculture, we observed cell fusion events similar to those seen in Fig. 3, where a red C. acetobutylicum-Halo was fused with a *C. ljungdahlii* cell containing labeled RNA (Fig. 7A). This pair in particular is interesting since the red C. acetobutylicum-Halo acquired some of *C. ljungdahlii*’s green RNA during the fusion, while the *C. ljungdahlii* cell did not acquire any red protein from C. acetobutylicum-Halo. This observation may suggest that the exchange of RNA between the two organisms occurs at a higher rate compared to protein exchange described earlier. Flow-cytometry analysis captured the kinetics of RNA exchange more accurately; double-positive cells were observed after 2 h of coculture, reaching 51.9% by hour 27 (Fig. S8). Figure 7B shows a cell fusion pair where the protein and RNA were fully exchanged and equally distributed in each cell, resulting in the formation of hybrid cells. Single hybrid cells—containing uniformly distributed green RNA and red protein material—were observed throughout the coculture at 4 h (Fig. 8A), 9 h (Fig. 7B), and 20 h (Fig. 8B). The cell of Fig. 8B, containing the red HaloTag from C. acetobutylicum and the green RNA acquired from *C. ljungdahlii*, is of particular interest: it is a cigar-shaped, swollen clostridial form cell, which is part of C. acetobutylicum’s morphogenetic program of sporulation (19, 21). It has all the requisite characteristics of such cells (see reference 16): the cigar shape (thick in the middle), the large size (typically 5.5 μm long by 1 μm wide in the middle, as is the case here), and nonuniform distribution of cellular material. The nonuniform cellular content is aptly displayed when one compares the images of Fig. 8A and 8B. The cell of Fig. 8A is a 2 μm long by 0.6 μm wide vegetative cell from hour 4 of the coculture. Thus, C. acetobutylicum that acquired foreign RNA from *C. ljungdahlii* was still able to function and go through its normal sporulation (differentiation) process. This is a persisting cell capable not only of cell division, but also of continuing the characteristic *Clostridium* sporulation program (18, 20). Based largely on the Escherichia coli model, the average half-life of mRNAs is 2 to 8 min (32, 33) during exponential growth, and twice that during stationary phase. If one assumes that *C. ljungdahlii* and C. acetobutylicum have similar mRNA half-lives, then the SYTO RNASelect imaging data of Fig. 7 and 8 are based on labeling of rRNA and tRNA, which are very stable and make up a very large fraction (>90%) of total RNA (34). We have tested the stability of the RNASelect-labeled RNA in C. acetobutylicum and *C. ljungdahlii*, and found the fluorescence to be stable for 20 h of culture (Fig. S9).

10.1128/mBio.02030-20.8 FIG S8 Flow-cytometric examination of a coculture between red C. acetobutylicum-Halo cells labeled with Janelia Fluor646 ligand, and WT *C. ljungdahlii* cells labeled green with SYTO RNASelect dye. The gates shown in Fig. S7 were used to examine this coculture. The percentages in each quadrangle represent the fraction of the total population in each gate. The numbers in parentheses represent the normalized fraction of fluorescent cells only, where each fluorescent fraction (green^+^, red^+^, double-positive) was divided by the total fluorescent fraction without counting the nonfluorescent cells in gate Q3-3. A significant number of double-positive cells (3.6%) was detected after 2 h of coculture, indicating fast RNA exchange. The fraction of double-positive cells increased to 51.9% at hour 27 of coculture, indicating that a large amount of RNA is exchanged between the two organisms. Download FIG S8, DOCX file, 1.5 MB. Copyright © 2020 Charubin et al. 2020 Charubin et al. This content is distributed under the terms of the Creative Commons Attribution 4.0 International license.

10.1128/mBio.02030-20.9 FIG S9 Testing the stability and labeling of the RNASelect dye in C. acetobutylicum and *C. ljungdahlii* monocultures. C. acetobutylicum and *C. ljungdahlii* were incubated with RNASelect dye for 1 h and then washed in fresh medium. The green fluorescence of the RNASelect-labeled cells was measured using flow cytometry for 20 h. After labeling (0 h), almost complete labeling was observed (97% in C. acetobutylicum and 94% in *C. ljungdahlii*). Over the course of 20 h, some cells lost the green fluorescence, most likely due to RNA breakdown, with the green fluorescent fraction dropping to 58% in C. acetobutylicum and 66% in *C. ljungdahlii*. Importantly, the green fluorescent fraction did not increase over time, which would indicate cells were using RNASelect-labeled nucleotides to produce new RNA at later time points. Download FIG S9, DOCX file, 0.7 MB. Copyright © 2020 Charubin et al. 2020 Charubin et al. This content is distributed under the terms of the Creative Commons Attribution 4.0 International license.

## DISCUSSION

Fusion events are necessary for the exchange of proteins and RNA between the two organisms (Fig. 3, Fig. 5A, Fig. 7). Fusion events apparently result in the formation of C. acetobutylicum-*C. ljungdahlii* hybrid cells (Fig. 4, Fig. 5B, Fig. 7B, and Fig. 8) which, once formed, continue to grow (Fig. 5B), apparently divide (Fig. 4), and, as displayed in Fig. 8B, continue the sporulation program. Given the exchange of proteins and RNA, one would assume the exchange of electron carrier small molecules (NADH) or proteins such as ferredoxins also takes place. However, the presence of hybrid cells that would share electron-transport proteins and other electron carriers makes the issue of electron exchange between the two organisms a moot point.

As we explained, the dynamics of protein exchange (Fig. 6 and Fig. S6) underestimate the extent of the cells with cellular material from both organisms. Labeled RNA is then a better measure of the large-scale exchange of cytoplasmic material; the 51.9% double-positive population observed after 27 h (Fig. S8) is indicative of the unprecedented level of interspecies cytoplasmic exchange, which, as we discuss below, has enormous fundamental and practical implications. One should note that even this large 51.9% fraction is a one-time snapshot of the population that underestimates the cumulative number of double-positive cells containing cytoplasmic material from both organisms. A better measure of the extent of the cytoplasmic material exchange is the very low fraction of the green-RNA-labeled *C. ljungdahlii* cells after 40 h of coculture. The loss of the green cells is not due to green RNA dilution as *C. ljungdahlii* cells grow slowly (compared to C. acetobutylicum), and in pure culture there is a minimal loss of green fluorescence. This means that by 40 h, most of the *C. ljungdahlii* cells are double-positive but are detected as red cells due to the dilution of green RNA in the faster-growing C. acetobutylicum cells, which keep expressing the red HaloTag protein.

In the Benomar et al. report (6), interspecies interactions were not polar, showing no preference for cellular topography at the contact point. Their images suggest the exchange of calcein and mCherry through visualized formation of cellular bridges of unknown nature. Their data suggest neither membrane and cell wall fusions, nor do they suggest massive protein or RNA exchange, or the persistence of hybrid cells. In contrast, we demonstrate that interspecies cell fusion is necessary for the massive exchange of cytoplasmic material. The cell-fusion-driven large-scale exchange of proteins and RNA reported here is distinct from the exchange of cellular material involving nanotubes (14–17), as there is no evidence of any nanotube structures here. Instead, it is the extensive fusion events of the cell walls and membranes at the cell poles that drive the exchange. In some cases, formation of nanotubes does not require nutritional stress or syntrophic interactions (15), but in other cases it does (14).

It is unlikely that the observed fusion and protein and RNA exchange events are unique to this syntrophic pair, as many such pairs exist in nature. The survival benefits of such events are clear, and of physiological significance. As such, fusion and cellular material exchange events are likely broadly distributed in microbiomes. The implications of the cell fusion and material exchange between organisms would be profound in population and evolutionary biology, antibiotic resistance, and microbiome behavior. Such fusion events lead to an expanded metabolic network of reactions beyond a simple sum of the metabolism of the two organisms—an expanded metabolic space—beyond what is possible in a single organism. Therefore, fusion events can be explored for synthetic biology applications based on sharing recombination machineries, restriction modification and methylation capabilities, CRISPR capabilities, mechanisms for resistance to antibiotics, toxic chemicals, damaging radiation, and acquisition of several other traits that different organisms possess.

Heterologous cell fusion may have a profound impact on unexplained microbiological phenomena. An example is antibiotic heteroresistance of pathogens, which lack antibiotic-resistance genes but display a resistance phenotype resulting in persistent infections (8). This could result from pathogens acquiring resistance proteins from nonpathogenic commensal bacteria via heterologous fusion. In another example, several pathogens can avoid immune responses and invade deep tissues by mechanisms that are not yet fully understood (35, 36). Fusion events leading to hybrid cells, even temporarily, may enable pathogens to get entry to host tissues by acquiring tissue-entry machinery they lack from a commensal organism, and/or camouflage themselves to avoid immune detection. Furthermore, since the bacteria that can be grown in the laboratory are only a small fraction of microbial diversity found in nature (10), heterologous cell fusion might explain the existence of at least some of the unculturable bacteria cases (10). This would be supported by noting that some of these organisms have been cultivated in the laboratory using cocultures mimicking the natural environment (10). It is not unlikely that some of these microbes have evolved their genome to exist only in a syntrophic state. Syntrophy could then be viewed as an alternate evolutionary trajectory for microbes, similar in principle to that of phage or virus evolutionary trajectory.

## MATERIALS AND METHODS

### Microorganisms and culture media.

Monocultures and cocultures of C. acetobutylicum (ATCC 824), C. acetobutylicum fluorescent strains (23) (C. acetobutylicum*-*FAST, C. acetobutylicum*-*ZapA-FAST, and C. acetobutylicum-Halo), *C. ljungdahlii* (ATCC 55383), and *C. ljungdahlii* fluorescent strain (*C. ljungdahlii*-Halo) were grown in Turbo CGM medium as previously described (13). Turbo CGM used for *C. ljungdahlii* monocultures was supplemented with 5 g/liter fructose, and these cultures were grown in serum bottles with 20 lb/in^2^g of H_2_/CO_2_ gas mixture (80/20%). Turbo CGM used for C. acetobutylicum monocultures and cococultures was supplemented with 5 g/liter fructose and 80 g/liter glucose, and these cultures were grown in unpressurized bottles in the anaerobic chamber.

### Construction of fluorescent *C. ljungdahlii*-Halo and C. acetobutylicum-Halo strains.

We constructed the p100ptaHalo plasmid expressing the HaloTag peptide, which can covalently bind to multiple fluorescent ligands (30, 31). The p100ptaHalo backbone was the modified pSOS95 clostridia-E. coli shuttle vector (Amp^R^, MLS^R^, *thl* promoter, rho-independent terminator, ColE1 ori, repB ori) (37). The modified pSOS95 backbone contains the repB origin of replication sequence (ori) to enable plasmid propagation in both C. acetobutylicum and *C. ljungdahlii*. The HaloTag operon was synthesized as a g-block by IDT, containing the P*_pta_* promoter from *C. ljungdahlii* (AAATGCCTAAGTGAAATATATACATATTATAACAATAAAATAAGTATTAGTGTAGGATTTTTAAATAGAGTATCTATTTTCAGATTAAATTTTTGCTTATTTGATTTACATTATATAATATTGAGTAAAGTATTGACTAGCAAAATTTTTTGATACTTTAATTTGTGAAATTTCTTATCAAAAGTTATATTTTTGAATGATTTTTATTGAAAAATACAACTAAAAAGGATTATAGTATAAGTGTGTGTAATTTTGTGTTAAATTTAA) (37), an optimized RBS sequence (GAGAGGAGGATTAGTC) (38), the codon-optimized HaloTag gene (ATGGCTGAAATTGGAACTGGATTCCCATTTGACCCTCATTACGTAGAGGTATTGGGTGAAAGGATGCACTACGTAGATGTAGGACCTAGAGACGGAACTCCTGTATTATTTTTGCACGGTAATCCAACAAGCTCATACGTTTGGAGGAACATAATACCTCATGTAGCACCAACTCACAGGTGTATAGCACCAGACTTAATTGGAATGGGAAAATCTGATAAACCTGATCTTGGATATTTCTTTGACGATCATGTTAGATTTATGGACGCATTTATTGAAGCTTTGGGTTTAGAGGAAGTTGTGCTAGTAATACACGATTGGGGTAGCGCATTAGGTTTTCACTGGGCAAAGAGAAACCCTGAAAGAGTGAAAGGTATTGCTTTTATGGAGTTCATTAGGCCAATTCCTACATGGGACGAGTGGCCTGAATTTGCAAGGGAAACATTTCAAGCATTTAGAACTACAGACGTTGGAAGAAAGCTAATAATAGATCAAAATGTATTCATTGAAGGAACTTTACCAATGGGTGTTGTAAGACCTTTGACAGAGGTAGAAATGGACCATTACAGAGAACCATTCCTAAACCCAGTGGACAGGGAGCCTTTGTGGAGATTCCCTAACGAACTTCCTATAGCTGGAGAACCTGCTAATATTGTTGCTCTTGTAGAGGAGTACATGGATTGGTTACACCAGAGTCCAGTACCAAAGCTATTGTTCTGGGGTACTCCAGGAGTGTTGATTCCTCCAGCAGAGGCAGCTAGACTTGCTAAGAGCCTTCCAAATTGCAAAGCTGTAGATATTGGTCCAGGACTAAACCTATTACAGGAGGACAACCCAGATTTAATAGGTTCTGAGATTGCAAGGTGTTATCAACTCTTGAGATTTCAGGTTAA), and a compatible terminator (GAGTTACCTTAAATGGTAACTC) (37). PCR was used to amplify the backbone, which removed the *thl* promoter and generated homology regions with the HaloTag g-block. The p100ptaHalo was assembled in E. coli using standard cloning techniques. The assembled p100ptaHalo plasmid was electroporated into *C. ljungdahlii* and C. acetobutylicum to yield the *C. ljungdahlii*-Halo and C. acetobutylicum-Halo strains, respectively, using previously reported protocols (37–39). The expression and functionality of the HaloTag in the *C. ljungdahlii*-Halo and C. acetobutylicum*-*Halo colonies was confirmed through flow cytometry and fluorescence microscopy (Fig. S5).

### Monocultures.

C. acetobutylicum frozen stocks were streaked onto 2×YTG (13) plates and cultured in Turbo CGM to generate seed cultures (13). *C. ljungdahlii* frozen stocks were inoculated into liquid Turbo CGM and passaged as needed to generate seed cultures (13). Seed cultures of the fluorescent clostridia strains (C. acetobutylicum-FAST, C. acetobutylicum-ZapA-FAST, C. acetobutylicum*-*Halo, and *C. ljungdahlii*-Halo) used for coculture experiments were grown using the same procedure, with the solid and liquid media supplemented with erythromycin (100 μg/ml) to maintain the plasmid DNA. Culture pH was adjusted to 5.2 after 12 h of growth with sterile deoxygenated 1 M NaOH to prevent acid death (13).

### Cocultures.

Cocultures of C. acetobutylicum and *C. ljungdahlii* were prepared as reported (13). Briefly, late-exponential phase C. acetobutylicum seed cultures (optical density at 600 nm [OD_600_] of 6.0 to 8.0) were mixed with exponential-phase *C. ljungdahlii* seed cultures (OD_600_ of 0.4 to 0.6). Seed cultures of fluorescent clostridia strains (C. acetobutylicum*-*FAST, *C. ljungdahlii*-ZapA-FAST, C. acetobutylicum*-*Halo, and *C. ljungdahlii*-Halo) were spun down at 5,000 rpm and washed once in fresh Turbo CGM medium to remove any residual erythromycin, before being used to prepare cocultures. Seed cultures were used to achieve a desirable starting population ratio (R) (13) of 1 to 3, which means that the cocultures started with an equal or excess number of *C. ljungdahlii* cells (13). Coculture experiments were performed in unpressurized static 100-ml glass bottles in an anaerobic chamber with a total liquid volume of 30 ml, unless otherwise specified.

### Cocultures in transwell plates.

Cocultures between C. acetobutylicum-ZapA-FAST and wild-type *C. ljungdahlii* labeled with the CellTracker Deep Red in transwell plates were prepared as reported (13). Briefly, C. acetobutylicum-ZapA-FAST and red-labeled *C. ljungdahlii* seed cultures were spun down at 5,000 rpm and washed twice in fresh Turbo CGM medium to remove residual erythromycin and excess Deep Red dye, respectively. The membrane used to separate the cells had a 100-nm pore size. To prepare separated cocultures, 800 μl of washed C. acetobutylicum-ZapA-FAST was placed in the bottom of the well. Next, the membrane insert was placed in the well, and 200 μl of washed red-labeled *C. ljungdahlii* was placed in the insert. Unseparated cocultures, pure C. acetobutylicum-Zap-FAST, and pure red-labeled *C. ljungdahlii* control cultures were prepared with the same amount of each seed culture used in the separated cocultures. The volume of control monocultures was adjusted to a final volume of 1 ml with fresh Turbo CGM containing fructose and glucose.

### Fluorescent protein labeling of cells.

To activate FAST fluorescence, samples of C. acetobutylicum FAST, C. acetobutylicum ZapA-FAST, and cocultures thereof were transferred to a 20 μM HMBR solution, as reported (23). When interacting with the small FAST protein, the HMBR ligand produces green fluorescence (λ_em_ = 541 nm) when excited with blue light (λ_ex_ = 480 nm) (23, 24). Red fluorescent wild-type (WT) *C. ljungdahlii* cells were generated using CellTracker Deep Red dye (Thermo Fisher Scientific), which produces far-red fluorescence (λ_em_ = 660 nm) when excited with red light (λ_ex_ = 630 nm). Each tube of Deep Red dye containing 15 μg of powdered dye was resuspended in 20 μl of dimethyl sulfoxide (DMSO) to produce 1 mM solution of the dye (1,000×). To label wild-type *C. ljungdahlii*, 90 μl of the dissolved dye was added to a 90-ml culture of *C. ljungdahlii*. Cells were incubated at 37°C with the dye for 1 h to allow the dye to bind to *C. ljungdahlii*’s intracellular proteins. Following labeling, cells were centrifuged at 5,000 rpm anaerobically and washed twice in Turbo CGM medium (supplemented with glucose and fructose) to remove any excess dye. Control experiments showed that there was no residual dye or fluorescent protein that leaked out of cells to produce false double-positive cells (Fig. S3). Washed and red-labeled *C. ljungdahlii* cells were used to prepare cocultures and control monocultures. WT C. acetobutylicum cells were stained with the CellTrace CFSE protein dye (Thermo Fisher Scientific), which produces green fluorescence (λ_em_ = 517 nm) when exposed to blue light (λ_ex_ = 492 nm). An aliquot (18 μl) of DMSO was added to each tube of CellTraceTM CFSE dye powder to produce a 5 mM solution (1,000×). To label WT C. acetobutylicum cells, 30 μl of dissolved dye was added to 30 ml of C. acetobutylicum culture. Cells were incubated at 37°C with the dye for 1 h to allow the dye to bind to C. acetobutylicum’s intracellular proteins. Following labeling, cells were centrifuged at 5,000 rpm anaerobically and washed twice in Turbo CGM medium (supplemented with glucose and fructose) to remove any excess dye. Cells were incubated in the fresh Turbo CGM for 20 min to deactivate any remaining free dye. Washed cells were used to prepare coculture with WT *C. ljungdahlii* cells labeled with the CellTracker Deep Red. To activate the HaloTag fluorescence, 1-ml aliquots of *C. ljungdahlii*-Halo, C. acetobutylicum*-*Halo, and cocultures were mixed with 1 μl of 200 μM Janelia Fluor 646, or TMR Direct (Promega, WI) ligand solution and incubated at 37°C for 30 min to allow the ligand to bind to the intracellular HaloTag. After the labeling, samples were gently spun down at 1,000 rpm to remove excess ligand and resuspended in fresh Turbo CGM. Janelia Fluor 646 produced far-red fluorescence (λ_em_ = 664 nm) when exposed to red light (λ_ex_ = 646 nm), while the TMR Direct produced orange fluorescence (λ_em_ = 578 nm) when exposed to green light (λ_ex_ = 552 nm). Coculture samples containing fluorescent C. acetobutylicum-ZapA-FAST and *C. ljungdahlii*-Halo were first labeled with Janelia Fluor 646 to activate *C. ljungdahlii*-Halo’s fluorescence, and were then placed in 20 μM HMBR solution to activate C. acetobutylicum-ZapA-FAST fluorescence. Control labeling experiments were performed where C. acetobutylicum-ZapA-FAST was labeled with HaloTag ligand and *C. ljungdahlii*-Halo was labeled with HMBR to ensure that each ligand interacted only with its target protein. No cross-reactivity was observed with either.

### Fluorescent RNA labeling of cells.

The cellular RNA in WT *C. ljungdahlii* cells was labeled with SYTO RNASelect RNA-specific dye (Thermo Fisher). RNASelect dye produces a strong green fluorescence (λ_em_ = 530 nm) only when bound to RNA molecules. To label WT *C. ljungdahlii* cells, 30 μl of the 5 mM SYTO RNASelect dye was added to 90 ml of growing *C. ljungdahlii* culture and incubated at 37°C for 1 h. After incubation, *C. ljungdahlii* cells were spun down and washed twice in Turbo CGM (supplemented with fructose and glucose) to remove any excess unbound dye. Green fluorescence of the RNASelect dye bound to RNA was detectable by flow cytometry and microscopy up to 30 h in pure *C. ljungdahlii* and coculture experiments. WT *C. ljungdahlii* cells labeled with RNASelect dye were used to prepare cocultures with the C. acetobutylicum-Halo strain to examine possible RNA exchange between the two organisms. Prior to microscopy or flow cytometry analysis, coculture samples were first labeled with Janelia Fluor 646 ligand, as described above, to label the HaloTag protein in the coculture sample.

### Superresolution structured illumination and confocal SR Airyscan microscopy.

To visualize the fluorescence of C. acetobutylicum and *C. ljungdahlii* cells in coculture, a 1-ml sample was collected, centrifuged at 3,000 rpm for 1 min, and resuspended in phosphate-buffered saline (PBS). Samples containing *C. ljungdahlii*-Halo or C. acetobutylicum*-*Halo were first labeled with the HaloTag ligand (as described above) before sample preparation for microscopy. Washed cells were placed in a Nunc Lab-Tek chamber slide (Thermo Fisher) coated with poly-l-lysine. Cells were incubated in the chamber for 1 h to immobilize cells and cell clusters on the poly-l-lysine coating. After 1 h, the chamber was rinsed with PBS thrice to remove excess cells, and filled with enough 20 μM HMBR solution to cover the surface of the chamber in order to prevent cell dehydration and activate the FAST protein. Coculture samples of C. acetobutylicum-Halo and *C. ljungdahlii* labeled with RNASelect dye were stored in sterile water instead of the HMBR solution. Immobilized cells were imaged using either an LSM 880 multiphoton confocal microscope (Carl Zeiss) with an Airyscan detector (which enables superresolution with a 2-fold increase in resolution), or Elyra PS.1 superresolution microscope (Carl Zeiss) capable of structured illumination microscopy (SR-SIM). Each sample was imaged using a 63×/1.4 oil objective to collect raw superresolution images, which were then processed in the Zen software (Carl Zeiss). A 488 nm blue laser was used to visualize green fluorescence of C. acetobutylicum-FAST, C. acetobutylicum-ZapA-FAST and wild-type *C. ljungdahlii* labeled with RNASelect dye. A 633 nm red laser was used to visualize red fluorescence of wild-type *C. ljungdahlii* labeled with Deep Red dye, and *C. ljungdahlii*-Halo and C. acetobutylicum-Halo strains labeled with the Janelia Fluor646 ligand.

### Flow cytometry.

Cells were analyzed using a BD FACSAria IIu flow cytometer (Becton, Dickinson). A blue solid-state laser (λ = 488 nm) and a 530/30 nm filter (FITC filter) was used to measure green fluorescence of the C. acetobutylicum-FAST, C. acetobutylicum-ZapA-FAST, and wild-type *C. ljungdahlii* labeled with RNASelect dye. A green solid-state laser (λ = 550 nm) and a 560/90 nm filter (PE filter) was used to measure orange fluorescence of HaloTag bound to TMR Direct ligand (C. acetobutylicum-Halo and *C. ljungdahlii*-Halo). A red solid-state laser (λ = 633 nm) and a 660/20 nm filter (APC filter) were used to measure the far-red fluorescence of the wild-type *C. ljungdahlii* cells labeled with CellTracker Deep Red dye, and HaloTag bound to Janelia Fluor 646 ligand (C. acetobutylicum-Halo and *C. ljungdahlii*-Halo). To measure fluorescence of monococultures or cocultures, 2 μl of cell suspension was transferred to 1 ml of 20 μM HMBR solution (to activate FAST) and were run through the flow cytometer. Samples containing the *C. ljungdahlii*-Halo or C. acetobutylicum-Halo strains were labeled with the HaloTag ligand beforehand as described above. Coculture samples of C. acetobutylicum-Halo and *C. ljungdahlii* labeled with RNASelect dye were placed in sterile water instead of HMBR solution. A total of 10,000 events were analyzed for each sample run. The delay time of each laser was adjusted appropriately to allow the simultaneous visualization of green, orange, and far-red signals.

### Transmission electron microscopy.

Cell cultures used for transmission electron microscopy (TEM) were fixed in 2% glutaraldehyde and 2% paraformaldehyde in 0.1 M sodium cacodylate buffer pH 7.4 and stored at 4°C until further processing. Fixed cells were pelleted, embedded in 4% low-melting-point agarose and cut into 1 mm^3^ cubes. Embedded cell pellets were washed in 0.1 M sodium cacodylate buffer, fixed in 1% osmium tetroxide in buffer, and then washed with Nanopure water. Samples were dehydrated with a graded series of acetone solutions and then transitioned from acetone to n-butyl glycidyl ether (n-BGE) to facilitate resin infiltration. Samples were gradually infiltrated with increasing concentrations of Quetol 651/NSA resin diluted with n-BGE (21). After several exchanges in 100% resin, samples were left overnight in 100% resin, and then embedded in BEEM capsules (21) and polymerized at 60°C for 24 h. Blocks were sectioned on a Reichert-Jung Ultracut E ultramicrotome (21) and ultrathin sections were collected onto 200 mesh Formvar-carbon coated copper grids. Sections were poststained with 2% uranyl acetate in 50% methanol and Reynolds’ lead citrate (21) and imaged with a Libra 120 transmission electron microscope (Carl Zeiss) operating at 120 kV. Images were acquired with an Ultrascan 1000 CCD camera (Gatan) using Digital Micrograph. All images were exported as uncompressed TIFF files.

### Electron tomography.

For electron tomography, sections were cut to 150 nm and placed on 200 mesh Formvar-carbon coated copper grids. Gold fiducial markers 10 nm and 15 nm in size were placed on either side of the section to assist with tomogram reconstruction. Sections were poststained as described above. Dual-axis tilt series were acquired on a Zeiss Libra 120 transmission electron microscope operating at 120 kV with a Gatan Ultrascan 1000 CCD camera using the Gatan Digital Micrograph tomography plugin. Tilt series from both axes were collected from either ±60° or ±65° with images recorded at every 1°. To collect a tilt series from the orthogonal direction, the grid was removed from the holder, manually rotated 90°, and the same region of interest was relocated. Tilt series were reconstructed and combined in IMOD (a computer software package used for analyzing 3D biological image data) using an r-weighted back-projection (40).

### Correlative fluorescence-TEM microscopy.

For correlative microscopy, 1-ml coculture samples were collected, centrifuged at 3,000 rpm for 1 min, and resuspended in PBS. Washed cells were placed onto specially prepared gridded coverslips to aid in reidentification of the cells and cell clusters imaged by fluorescence microscopy for ultramicrotomy. Briefly, an alphanumeric grid pattern was applied to the surface of the coverslip by using an SEM finder grid (Electron Microscopy Sciences, cat. no. 80101-Cu) as a mask and sputter-coating an 8-nm layer of platinum using a Leica EM ACE 600 (Leica Microsystems). A drop of 0.1% poly-l-lysine solution was placed on the sputter-coated surface of the coverslip, incubated for 15 min, and washed with water. Once the coverslip dried, 200 μl of the washed cells was placed on the coverslip, incubated for 40 min, and washed with PBS to remove any unbound bacteria. The coverslip was then immersed in a petri dish containing 20 μM HMBR solution to activate the FAST peptide. The coverslips were then imaged using Elyra PS.1 superresolution microscope (Carl Zeiss) capable of structured illumination microscopy (SR-SIM). Each grid was first imaged with a 10×/0.3 air objective to create a 10× map of the alphanumeric grid. Next, the sample was imaged using a 63×/1.4 oil objective to locate areas on the grid with C. acetobutylicum*-C. ljungdahlii* clusters, or cells displaying two fluorescent signals, and superresolution images were taken of each cluster. Once all regions of interest were imaged, the area was marked on the 10× map for identification during TEM analysis. Following fluorescent imaging, coverslips were processed for TEM as described above with the exception that after dehydration into 100% acetone, EMBed-812 resin was used for resin infiltration and embedding. For embedment, excess resin was drained from the coverslip, and the backside was wiped clean. The coverslip was then placed on a microscope slide, sample-side up. A BEEM capsule with the lid removed and pointed tip cut off was placed over the region mapped by fluorescence microscopy, and this assembly was polymerized overnight at 60°C. The BEEM capsule was then filled with resin and polymerized an additional 24 h at 60°C. To remove the embedded bacteria from the glass coverslip, the polymerized slide-coverslip assembly was placed on a warm hotplate until the resin softened. A razor blade was used to score around the BEEM capsule, and then by applying lateral pressure to the BEEM capsule, the BEEM capsule was separated from the glass coverslip. The alphanumeric pattern left by the sputter-coating was imprinted on the freshly exposed surface of the BEEM capsule, which allowed the same region of interest imaged by fluorescence microscopy to be identified in the ultramicrotome. Ultrathin serial sections were collected using a Reichert-Jung Ultracut E ultramicrotome (21) and picked up onto 2 × 1 copper slot grids, which were then dried on a domino rack (Electron Microscopy Sciences, cat. no. 70621) that had been previously coated with a film of 0.5% Formvar in ethylene dichloride. The grids were poststained and imaged as described in the previous section describing TEM preparation and imaging.
