# Supplementary material for: Interspecies Microbial Fusion and Large-Scale Exchange of Cytoplasmic Proteins and RNA in a Syntrophic Clostridium Coculture
Source: mBio. 2020 Sep 1;11(5):e02030-20. doi: 10.1128/mBio.02030-20 (PMC7468208; doi:10.1128/mBio.02030-20)

**Fig S1. Cell fusion between C. acetobutylicum (Cac) and C. ljungdahlii (Clj) in the coculture.** Additional TEM images of the Cac-Clj fusion after 24 hours of coculture. Cocultures contained sporulating Cac cell (with electron-translucent regions) and vegetative Clj cells (homogenous electron-dense cytoplasm. In each shown example, Clj appears to invade Cac’s cytoplasm, which leads to fusion of cell membrane and cell walls of both organisms. Red arrows indicate close-up (panels **B, D, F**) images of the fusion events of panels **A, C, E**, respectively.


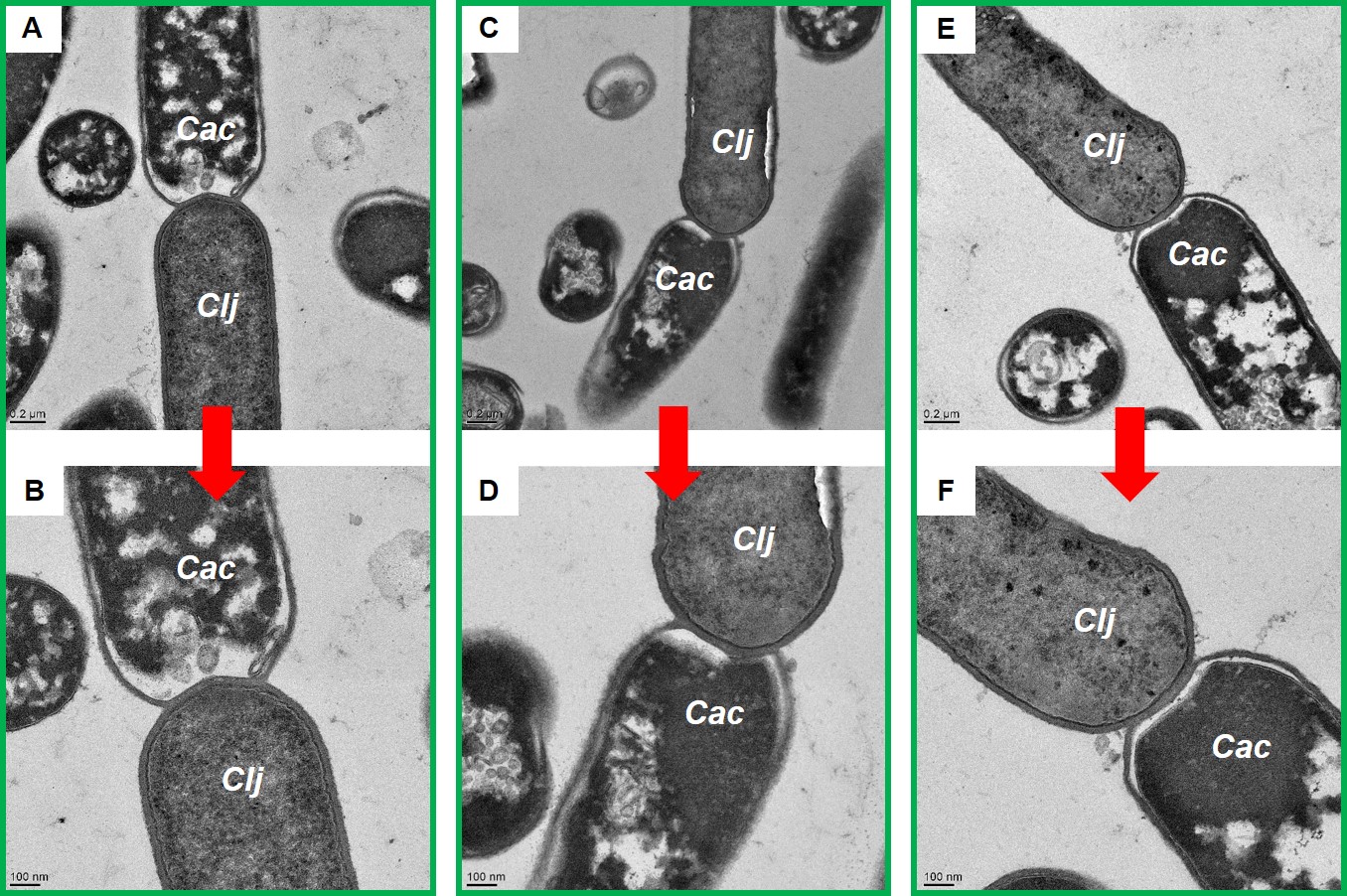

Supplement: FIG S1 [file mBio.02030-20-sf001.docx]
