# Supplementary material for: Interspecies Microbial Fusion and Large-Scale Exchange of Cytoplasmic Proteins and RNA in a Syntrophic Clostridium Coculture
Source: mBio. 2020 Sep 1;11(5):e02030-20. doi: 10.1128/mBio.02030-20 (PMC7468208; doi:10.1128/mBio.02030-20)

**Fig S3. Control experiments to confirm that protein exchange occurs only through Cac-Clj fusion.** (**A**) Experimental set up. Monocultures of Cac-ZapA-FAST and Red Clj (Deep Red dye) were grown for 24 hours. Entire cultures were pelleted and supernatants filtered with a 0.2 μm filter. ‘Red’ medium was the filtered spent medium from Red Clj culture; ‘Green’ medium was the filtered spent medium from Cac-ZapA-FAST culture. The Red Clj pellet was placed in the ‘Green’ medium, while the Cac-ZapA-FAST pellet was placed in the ‘Red’ medium. Cells in spent media were monitored using flow cytometry for 24 hours to detect if any fluorescent material that may have leaked into the spent media is possibly taken up by the other organism. (**B**) Flow cytometry analysis of cultures described in (**A**). Red Clj cells placed in ‘Green’ medium did not develop any green fluorescence after 24 hours in culture. Cac-ZapA-FAST cells placed in ‘Red’ medium did not develop any red fluorescence after 24 hours of culture. Therefore, there was no fluorescent material in the spent media that could be up taken by cells to produce false double-positive cells. Thus, protein exchange can only occur through Cac-Clj fusion.


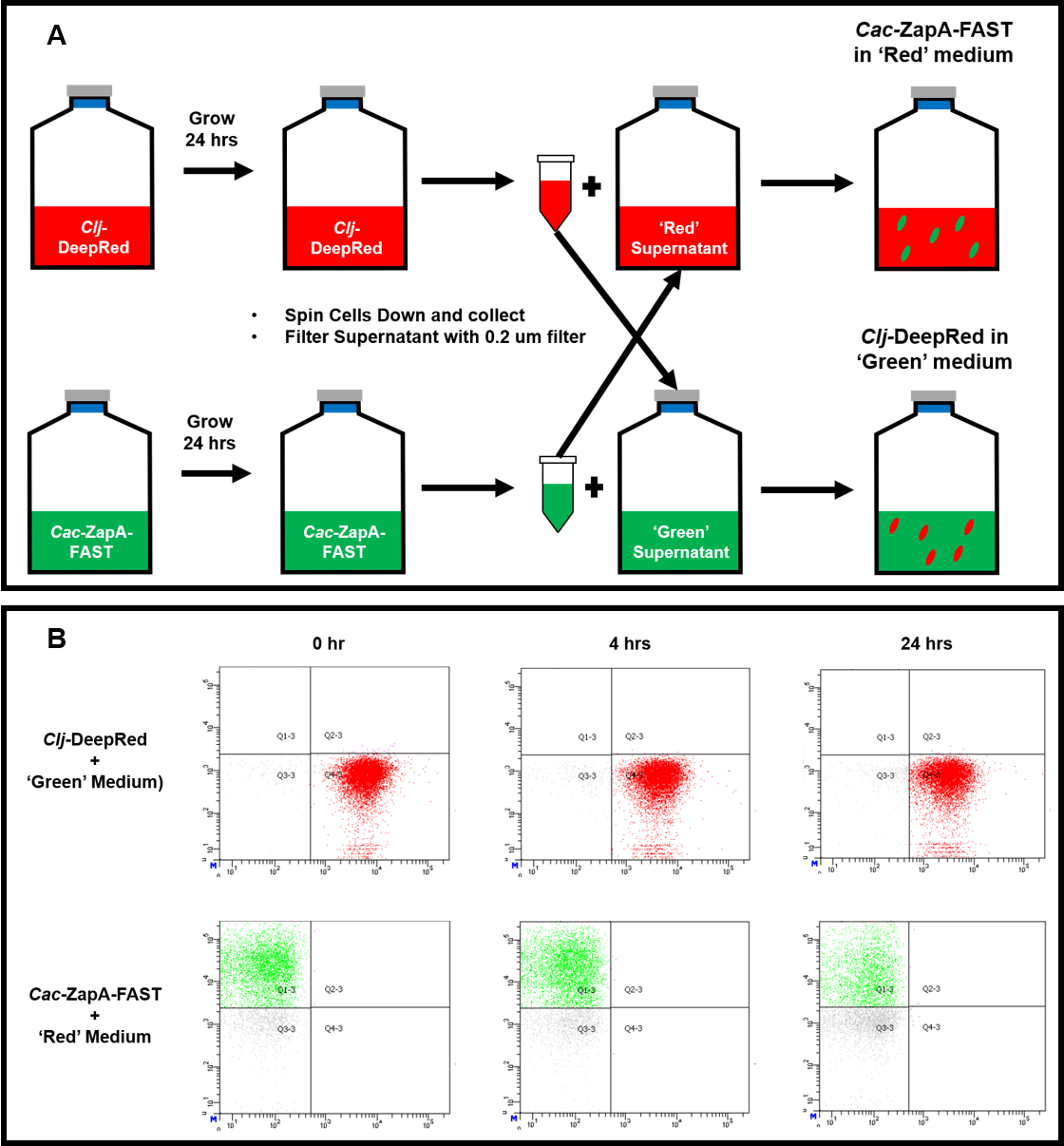

Supplement: FIG S3 [file mBio.02030-20-sf003.docx]
