# Supplementary material for: Interspecies Microbial Fusion and Large-Scale Exchange of Cytoplasmic Proteins and RNA in a Syntrophic Clostridium Coculture
Source: mBio. 2020 Sep 1;11(5):e02030-20. doi: 10.1128/mBio.02030-20 (PMC7468208; doi:10.1128/mBio.02030-20)
Supplement: FIG S4 [file mBio.02030-20-sf004.docx]

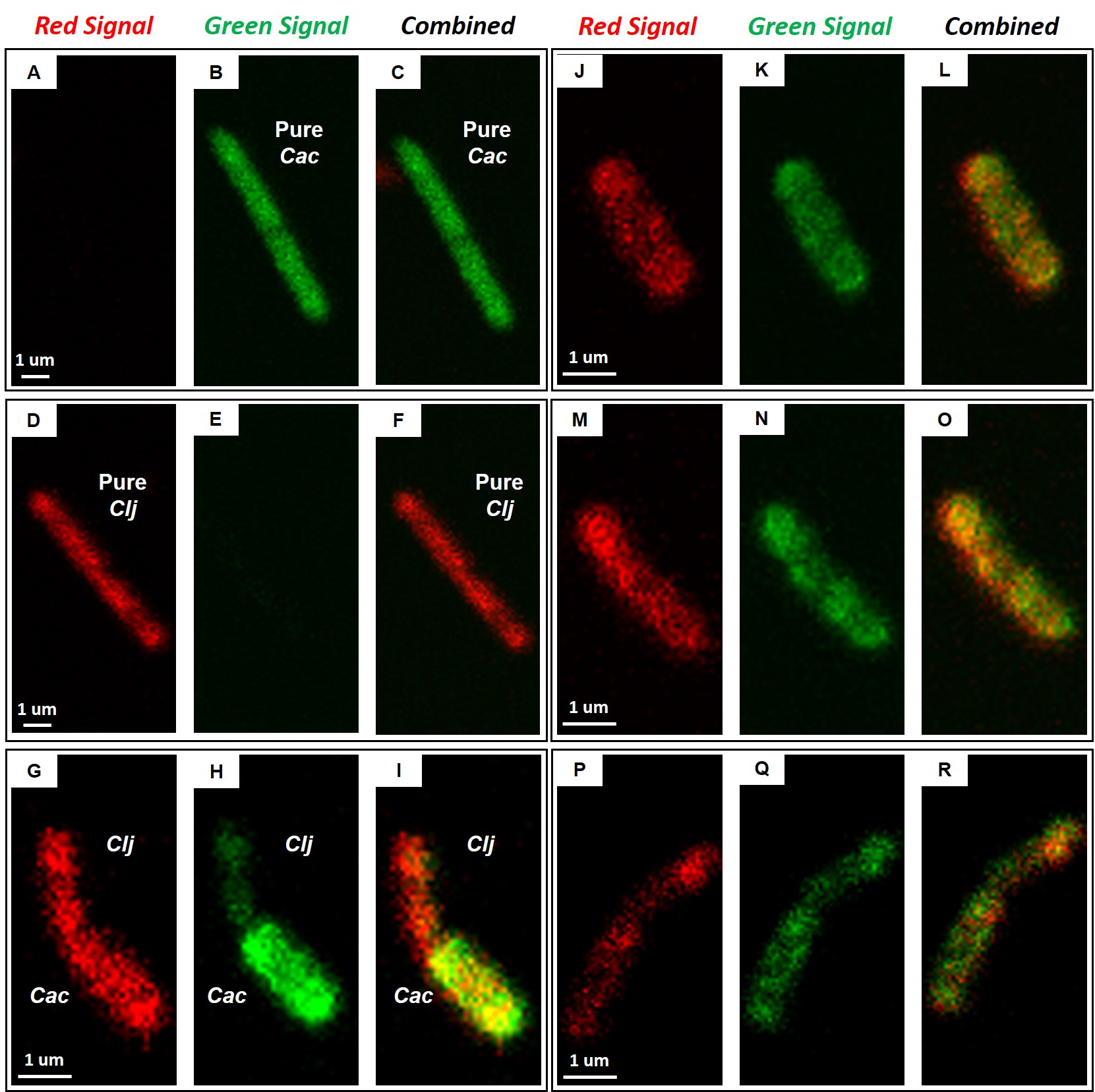


**Fig S4. Protein exchange after 20 hours in coculture between red-labeled WT Clj (labeled with CellTracker^TM^ Deep Red) and green-labeled WT Cac (labeled with CellTrace^TM^ CFSE)**. Images acquired by SR Airyscan confocal microscopy. Many cells exchanged proteins as assessed by exchange of green or red-labeled proteins, but several did not. (**A-C**) Single long green “pure” Cac cell, probably undergoing cell division. (**D-F**) A long red “pure” Clj cell probably undergoing cell division. (**G-I**) Pairs of cells similar to those of Fig. 1 and Fig. 3 of main text, where a Red Clj cell fused with a green Cac cell, thus exchanging proteins. We also observed several double-positive hybrid cells containing equally distributed fluorescent signals at different cell cycle stages. (**J-L**) Single hybrid cell; (**M-O**) Elongated hybrid cell; (**P-R**) Long hybrid cell, undergoing cell division.
