# Supplementary material for: Interspecies Microbial Fusion and Large-Scale Exchange of Cytoplasmic Proteins and RNA in a Syntrophic Clostridium Coculture
Source: mBio. 2020 Sep 1;11(5):e02030-20. doi: 10.1128/mBio.02030-20 (PMC7468208; doi:10.1128/mBio.02030-20)

**Fig S5. Clj-Halo and Cac-Halo strains expressing the HaloTag protein used for fluorescent labeling of cells in the coculture.** (**A**) Fluorescent labeling of WT Clj cells with the HaloTag TMR Direct^TM^ ligand. (**B**) Fluorescent labeling of Clj-Halo strain with same ligand. (**D**) Fluorescent labeling of WT Cac with the same ligand. (**E**) Fluorescent labeling of Cac-Halo strain with the same ligand. Orange fluorescence was measured by flow cytometry. WT Clj and Cac cells do not fluoresce after labeling with TMR Direct^TM^ ligand. Clj-Halo and Cac-Halo strains show high intensity orange fluorescence when labeled with TMR Direct^TM^ ligand. (**C**) SR-SIM imaging of Clj-Halo strain labeled with the far-red Janelia Fluor® 664 ligand. (**F**) SR-SIM imaging of Cac-Halo strain labeled with Janelia Fluor® 664 ligand. The Clj-Halo and Cac-Halo cells showed strong far-red fluorescence.


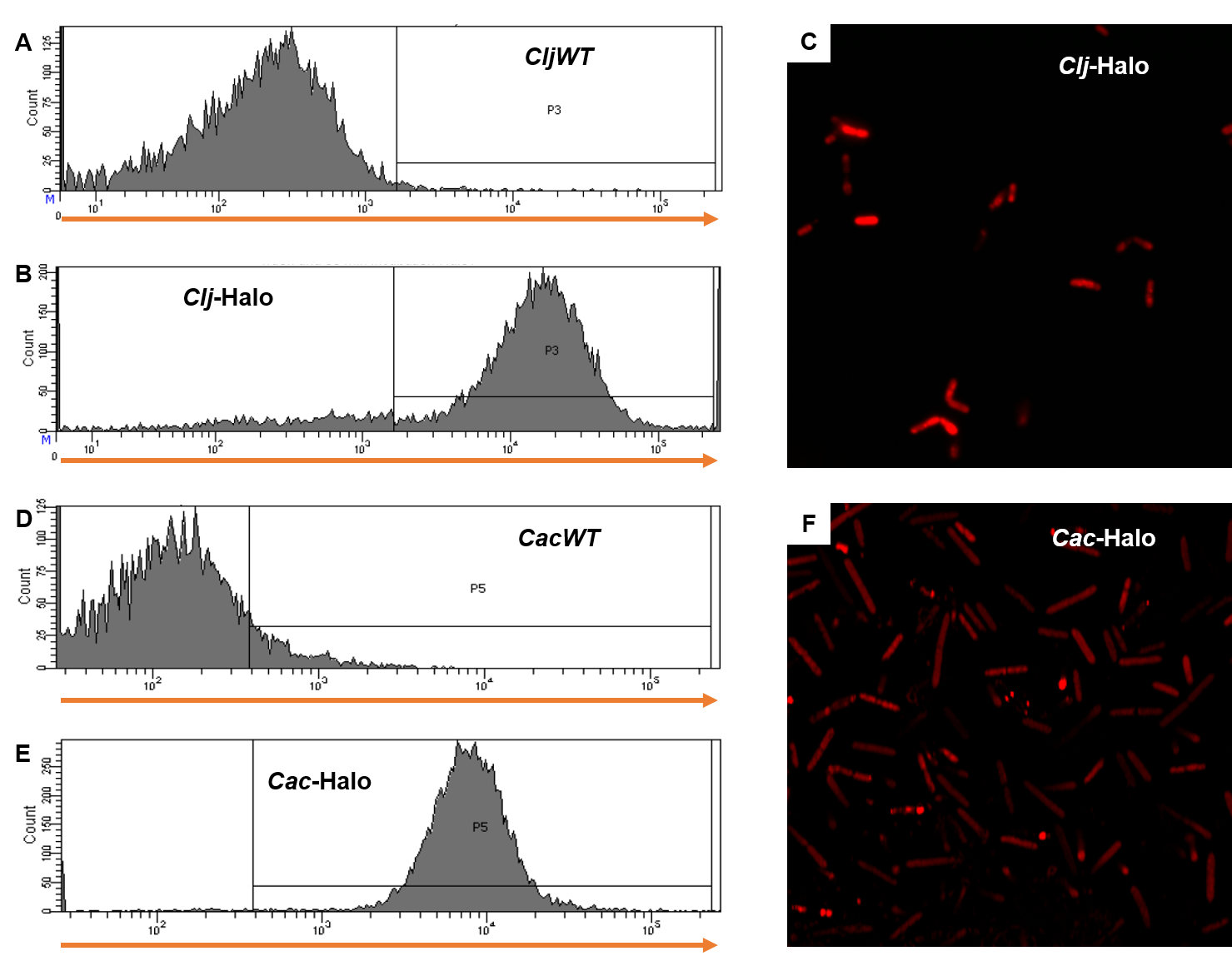

Supplement: FIG S5 [file mBio.02030-20-sf005.docx]
