# Supplementary material for: Interspecies Microbial Fusion and Large-Scale Exchange of Cytoplasmic Proteins and RNA in a Syntrophic Clostridium Coculture
Source: mBio. 2020 Sep 1;11(5):e02030-20. doi: 10.1128/mBio.02030-20 (PMC7468208; doi:10.1128/mBio.02030-20)

**Fig S8. Flow-cytometric examination of a coculture between red Cac-Halo cells labeled with Janelia 646 ligand, and WT Clj cells labeled green with SYTO^TM^ RNASelect^TM^ dye.** The gates shown in Fig. S7 were used to examine this coculture. The percentages in each quadrangle represent the fraction of the total population in each gate. The numbers in parentheses represent the normalized fraction of fluorescent cells only, where each fluorescent fraction (green^+^, red^+^, double-positive) was divided by the total fluorescent fraction without counting the nonfluorescent cells in gate Q3-3. A significant number of double-positive cells (3.6%) were detected after 2 hours of coculture indicating fast RNA exchange. The fraction of double-positive cells increased to 51.9% at hour 27 of coculture, indicating that a large amount of RNA is exchanged between the two organisms.


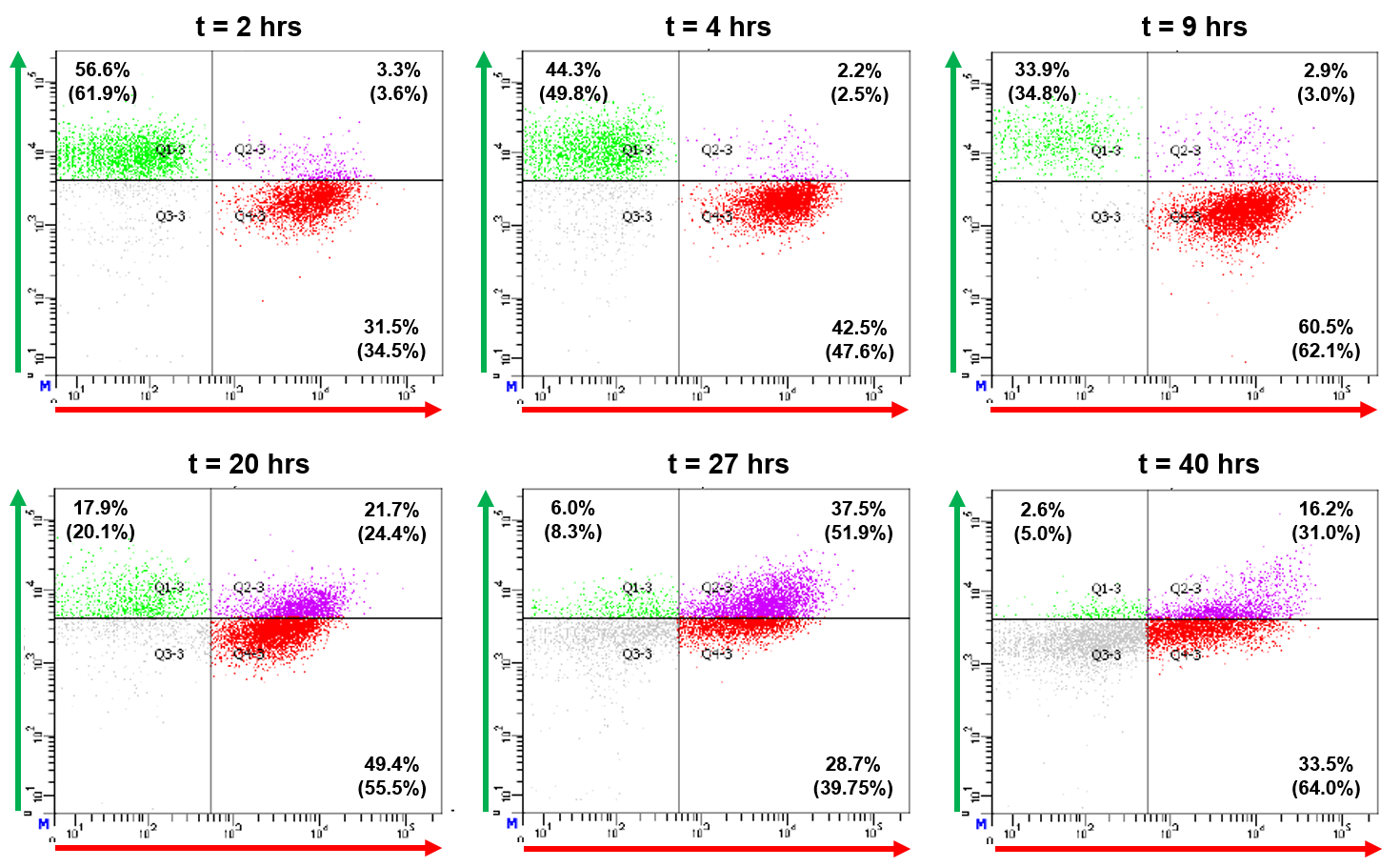

Supplement: FIG S8 [file mBio.02030-20-sf008.docx]
