# Supplementary material for: Interspecies Microbial Fusion and Large-Scale Exchange of Cytoplasmic Proteins and RNA in a Syntrophic Clostridium Coculture
Source: mBio. 2020 Sep 1;11(5):e02030-20. doi: 10.1128/mBio.02030-20 (PMC7468208; doi:10.1128/mBio.02030-20)
Supplement: FIG S9 [file mBio.02030-20-sf009.docx]

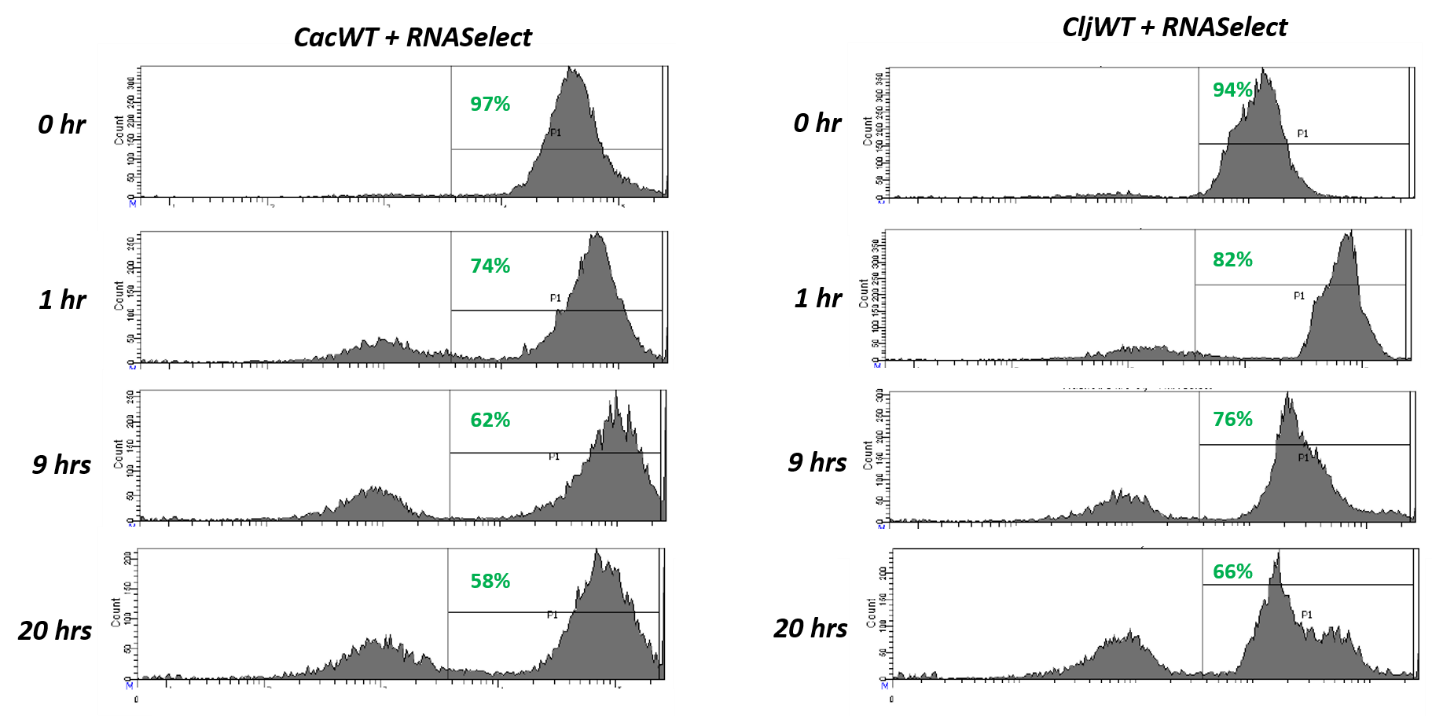


**Fig S9. Testing the stability and labeling of the RNASelect dye in Cac and Clj monocultures.** Cac and Clj were incubated with RNASelect dye for 1 hour and then washed in fresh medium. The green fluorescence of the RNASelect-labeled cells was measured using flow cytometry for 20 hours. After labeling (0 hours) almost complete labeling was observed (97% in Cac and 94% in Clj). Over the course of 20 hours, some cells lost the green fluorescence, most likely due to RNA breakdown, with the green fluorescent fraction dropping to 58% in Cac and 66% in Clj. Importantly, the green fluorescent fraction did not increase over time, which would indicate cells were using RNASelect-labeled nucleotides to produce new RNA at later time points.
