# Supplementary material for: Interspecies Microbial Fusion and Large-Scale Exchange of Cytoplasmic Proteins and RNA in a Syntrophic Clostridium Coculture
Source: mBio. 2020 Sep 1;11(5):e02030-20. doi: 10.1128/mBio.02030-20 (PMC7468208; doi:10.1128/mBio.02030-20)
Supplement: FIG S2 [file mBio.02030-20-sf002.docx]

**Fig S2. TEM imaging of C. ljungdahlii (Clj) and C. acetobutylicum (Cac) cell division in monocultures.** TEM images of vegetative Clj cells undergoing cell division. (**A**) Pinching at the midpoint to begin cell division. (**B**) Beginning of septum formation. (**C**) Septum is fully formed. (**D**) Daughter cells fully identifiable with distinct cell membranes and walls. No cytoplasmic invasion is present, as compared to cell fusion events of Fig. 1 and Fig. S1 in the coculture. (**E-F**) Cac cell division undergoes in the same fashion and does not resemble cell fusion observed in the coculture.
