# Supplementary material for: Interspecies Microbial Fusion and Large-Scale Exchange of Cytoplasmic Proteins and RNA in a Syntrophic Clostridium Coculture
Source: mBio. 2020 Sep 1;11(5):e02030-20. doi: 10.1128/mBio.02030-20 (PMC7468208; doi:10.1128/mBio.02030-20)
Supplement: FIG S6 [file mBio.02030-20-sf006.docx]

**Fig S6. Flow-cytometric examination of a coculture between green Cac-ZapA-FAST cells and wild-type (WT) Clj cells labeled with CellTracker^TM^ Deep Red.** Green (vertical) and red (horizontal) axes represent the intensity of green and red fluorescence. Gates for green (Q1-3), red (Q4-3), and double-positive (Q2-3) quadrangles (Q) were set based on fluorescence of individual strains and unlabeled cells (Fig. S7). Percentages represent the fraction of the total cell population in each quadrangle. Numbers in parentheses represent the normalized fraction of fluorescent cells only, obtained by dividing the cells in each fluorescent population by the total number of fluorescent cells. Unlabeled cells were the result of fluorescent cells with a signal too weak to detect. At one hour, there was an equal number of green Cac-ZapA-FAST (49.2%) and red-labeled Clj (48.7%) with few double-positive cells. The two organisms appear to form many fusion events (double-positive cells) during the first 11 hours reaching 17.5% of the population. The fraction of double positive cells decreased after 11 hours and disappeared after 27 hours.
