# Supplementary material for: Interspecies Microbial Fusion and Large-Scale Exchange of Cytoplasmic Proteins and RNA in a Syntrophic Clostridium Coculture
Source: mBio. 2020 Sep 1;11(5):e02030-20. doi: 10.1128/mBio.02030-20 (PMC7468208; doi:10.1128/mBio.02030-20)
Supplement: FIG S7 [file mBio.02030-20-sf007.docx]

**Fig S7. Setting flow cytometry gates for analysis of cocultures between Cac-ZapA-FAST and WT Clj labeled with CellTracker^TM^ Deep Red**. Gates 1-3, 2-3, 3-3, and 4-3 contain green, double-positive (labeled), unlabeled, and red cells, respectively. (**A-B**) Gate 3-3 for unlabeled cells was set using WT Clj cells with no ligand and the HMBR ligand (for the FAST protein), respectively. (**C**) Gate 4-3 for pure red cells was set using pure Clj cells labeled with CellTrackerTM DeepRed. (**D**) Gate 1-3 for pure green cells was set using pure Cac-ZapA-FAST cells mixed with HMBR ligand. Accordingly, gate 2-3 contains any cells that acquired both fluorescent proteins. The green (vertical) and red (horizontal) axes represent the intensity of green and red fluorescent signals, respectively. Green Cac-ZapA-FAST cells are not 100% labeled due to low fluorescence of a cell subpopulation, as has been documented (23).
